# Phage spanins: diversity, topological dynamics and gene convergence

**DOI:** 10.1186/s12859-018-2342-8

**Published:** 2018-09-15

**Authors:** Rohit Kongari, Manoj Rajaure, Jesse Cahill, Eric Rasche, Eleni Mijalis, Joel Berry, Ry Young

**Affiliations:** 1 Center for Phage Technology, Department of Biochemistry and Biophysics, Texas A&M University, 2128 TAMU, College Station, TX 77843-2128 USA; 2 National Institutes of Health, Bethesda, MD USA; 3 University of California, San Francisco, CA USA

**Keywords:** Bacteriophage lysis, Spanins, Membrane fusion, Gene evolution, Secondary structure predictions, Genetic architecture, Intermolecular disulfide bonds, Lipoproteins

## Abstract

**Background:**

Spanins are phage lysis proteins required to disrupt the outer membrane. Phages employ either two-component spanins or unimolecular spanins in this final step of Gram-negative host lysis. Two-component spanins like Rz-Rz1 from phage lambda consist of an integral inner membrane protein: i-spanin, and an outer membrane lipoprotein: o-spanin, that form a complex spanning the periplasm. Two-component spanins exist in three different genetic architectures; embedded, overlapped and separated. In contrast, the unimolecular spanins, like gp*11* from phage T1, have an N-terminal lipoylation signal sequence and a C-terminal transmembrane domain to account for the topology requirements. Our proposed model for spanin function, for both spanin types, follows a common theme of the outer membrane getting fused with the inner membrane, effecting the release of progeny virions.

**Results:**

Here we present a SpaninDataBase which consists of 528 two-component spanins and 58 unimolecular spanins identified in this analysis. Primary analysis revealed significant differences in the secondary structure predictions for the periplasmic domains of the two-component and unimolecular spanin types, as well as within the three different genetic architectures of the two-component spanins. Using a threshold of 40% sequence identity over 40% sequence length, we were able to group the spanins into 143 i-spanin, 125 o-spanin and 13 u-spanin families. More than 40% of these families from each type were singletons, underlining the extreme diversity of this class of lysis proteins. Multiple sequence alignments of periplasmic domains demonstrated conserved secondary structure patterns and domain organization within family members. Furthermore, analysis of families with members from different architecture allowed us to interpret the evolutionary dynamics of spanin gene arrangement. Also, the potential universal role of intermolecular disulfide bonds in two-component spanin function was substantiated through bioinformatic and genetic approaches. Additionally, a novel lipobox motif, AWAC, was identified and experimentally verified.

**Conclusions:**

The findings from this bioinformatic approach gave us instructive insights into spanin function, evolution, domain organization and provide a platform for future spanin annotation, as well as biochemical and genetic experiments. They also establish that spanins, like viral membrane fusion proteins, adopt different strategies to achieve fusion of the inner and outer membranes.

**Electronic supplementary material:**

The online version of this article (10.1186/s12859-018-2342-8) contains supplementary material, which is available to authorized users.

## Background

It has long been thought that holin-endolysin function was necessary and sufficient to effect bacteriophage lysis and achieve liberation of progeny virions [1–4], except in phages of the mycolata, where a third functional class, the Lysin B esterases, are essential to degrade the waxy outer membrane [5, 6]. Recently, however, another functional class of proteins, the spanins, have been shown to be required for disruption of the outer membrane (OM) in Gram-negative hosts [7–10]. To establish a context for spanin function, a brief overview of the holin-endolysin pathway is required.

### Holin-endolysin lysis pathways

**Fig. 1:**
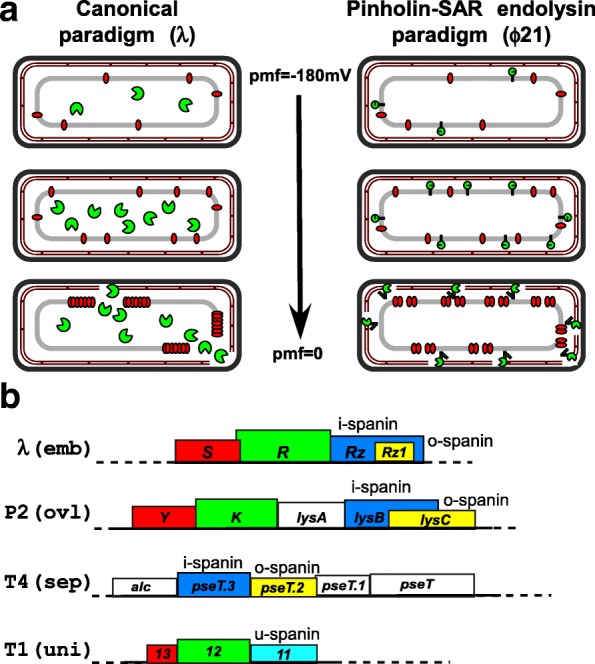
**a**: Cartoon representation of the canonical and “Pinholin-SAR endolysin” lysis paradigms of phages λ and ϕ21. The cartoon series begins with initial late gene expression and progresses downwards towards lysis as shown by the arrow. The cellular envelope components IM, PG and OM of a Gram-negative bacterial cell are shown as grey rectangle, hatched brown rectangle and black rectangle, respectively. In the canonical lysis paradigm (left), the holin (red ovals) accumulates in the IM while the active endolysin (open green symbols) accumulates in the cytoplasm. In the “Pinholin-SAR endolysin” lysis paradigm as in the phage ϕ21 (right), the SAR endolysins (closed green symbols) accumulate in the IM in an inactive form, anchored by a weakly hydrophobic TMD (green stub) alongside the pinholins (red ovals). See introduction for details on the pathways. **b**: Different spanin genetic architectures from the phages λ (embedded), P2 (overlapped), T4 (separated) and T1 (unimolecular). The rectangles, drawn to scale and labeled with appropriate names are color-coded to represent different lysis genes in each phage: red (holin), green (endolysin), blue (i-spanin), yellow (o-spanin) and cyan (u-spanin), while the uncolored rectangles represent genes of unrelated or unknown function. The spanin genes are also labeled with their spanin type on top of the gene in each case to highlight the genetic architecture. Note that color does not indicate sequence similarity; in fact, among all the genes depicted, only the K and R endolysin genes share any detectable similarity

In the canonical version of phage lysis (Fig. 1a) represented by phage λ, the endolysin, encoded by gene *R* (Fig. 1b), accumulates in the cytosol as fully-folded, active enzyme. Access to its substrate, the peptidoglycan (PG), is controlled by the holin, encoded by gene *S*. The holin gene product S105 accumulates harmlessly in the host inner membrane (IM) until, at a time programmed into its primary structure, suddenly “triggering” after reaching a critical concentration [3, 11]. Triggering is correlated with a sudden redistribution of the holin molecules in the IM to two-dimensional aggregates, referred to as “rafts”, and a collapse in the proton motive force (pmf). Upon triggering, lethal membrane lesions occur in the IM within these rafts, in the form of micron scale holes, the boundaries of which are lined by the two of the three transmembrane domains (TMDs) of S105 [12]. This breach in the IM allows the endolysins to escape the cytoplasm and attack the PG (Fig. 1a).

A second pathway to PG degradation, designated as the pinholin-SAR endolysin paradigm, has recently been described in detail, using the lambdoid phage ϕ21 (Fig. 1a) as a model [4]. In this pathway, the muralytic enzyme, R^21^, is secreted through the host translocon and accumulates in an inactive form in the periplasm tethered to the IM by an N-terminal TMD [4]. The membrane-tethered state of R^21^ requires the host pmf, so when the host membrane becomes de-energized, the TMD exits the bilayer, resulting in re-folding of the endolysin to its enzymatically active form, which then attacks the PG [13]. R^21^ has been designated as a SAR endolysin (Signal Anchor Release) to highlight the dynamic membrane topology. Control of R^21^ is exerted by the pinholin, S^21^68, a product of the ϕ21 *S* gene. Like S105, S^21^68 accumulates harmlessly as a homodimer uniformly distributed in the IM until triggering [11, 14]. Unlike the canonical holins, however, pinholins form ~ 10^3^ small (~ 2 nm) heptameric “pinholes”, which results in the depolarization of the bilayer and activation of the SAR endolysin [4, 13].

### Origin and characterization of the prototype spanins

For both lysis pathways, it has recently been shown that the spanins, encoded by the *RzRz1* genes in both lambda and ϕ21, are also required [9]. The lambda *RzRz1* genes had originally attracted attention because of their bizarre architecture, with *Rz1* embedded in the + 1 reading frame of *Rz* (Fig. 1b). Knockouts of either gene caused an absolute lysis defect, in which the host cells were converted into fragile spherical forms bounded by the intact OM [7]. The importance of these genes for lysis had been previously overlooked because in the context of the shaker flask, the fragile spherical cells were destroyed by shearing forces [9]. Thus, in cultures aerated by shaking, spanins are not required for lysis unless the OM is stabilized by addition of millimolar levels of divalent cations to the media. One surprising implication of these findings was that the OM alone could withstand the internal osmotic pressure in the absence of the PG layer, a conclusion also reached in studies of antibiotic-treated *E. coli* cells [15].

**Fig. 2:**
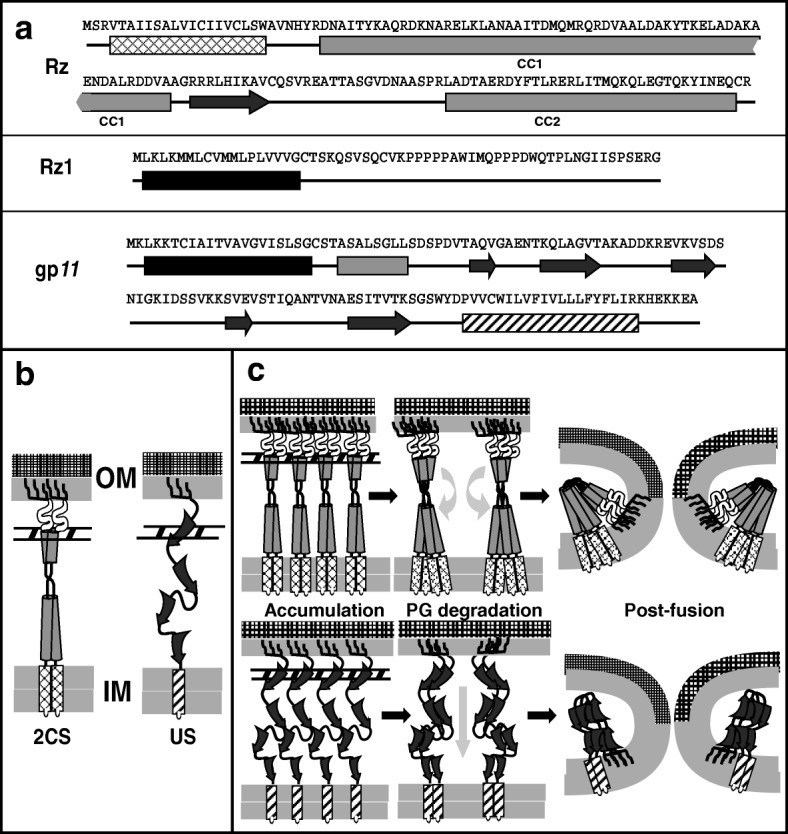
**a**: Predicted secondary structures of lambda i-spanin Rz, o-spanin Rz1 and T1 u-spanin gp*11*. The TMDs in Rz and gp*11* are shown as crossed and hatched rectangles respectively, while the lipoylation signal sequences in Rz1 and gp*11* are shown as black rectangles. Grey rectangles and black arrows indicate predicted alpha helical and beta sheet domains respectively. Coiled-coil domains CC1 and CC2 are connected through a flexible linker region. **b**: Cartoon representation of the topology of two-component spanin prototype from phage λ and the u-spanin from phage T1. In phage λ, i-spanin Rz is embedded to the IM by an N-terminal TMD (crossed rectangle) and has a periplasmic domain that constitutes two alpha helices (grey cylinders) connected by a linker, predicted to form coiled coils. The o-spanin Rz1 is attached to the inner leaflet of the OM via three fatty acyl chains (black lines) and has a periplasmic domain (white) predicted to be unstructured. The i- and o- spanins interact through their C-termini to form the spanin complex, linking the IM and OM though the PG meshwork. In T1, the u-spanin gp*11* is attached to the inner leaflet of the OM, by the three fatty acyl chains (black lines) at the N-terminus and to the inner membrane, through the C-terminal TMD (hatched rectangle). The periplasmic domain of gp*11*, predicted to be mainly extended beta sheets (black arrows), connects the IM and OM through the PG meshwork. **c**: Cartoon representation of the model for the function of two-component (top panels) and unimolecular (bottom panels) spanins. The spanin complexes accumulate within the constraints of the PG meshwork, connecting the IM and OM (left panels). Once the PG is removed by the endolysin, these complexes are free to undergo lateral diffusion and conformational changes (middle panels). These events bring both the membranes together, leading to fusion of the IM and OM (right panels) and release of the phage progeny through the fusion pore. The model presented in the top panel has already been presented in a previous report by Rajaure et al. [10]

Biochemical and genetic studies revealed Rz to be a class II (N-in, C-out) membrane protein in the IM, with a periplasmic domain dominated by predicted alpha helical domains (Fig. 2a, b) [8, 16, 17]. The alpha helices have a high propensity to form coiled-coils resulting in the periplasmic domain of Rz to be essentially divided into two coiled-coil domains, CC1 and CC2, connected by a flexible linker region (Fig. 2a). Rz1 was shown to be an OM lipoprotein lacking any detectable secondary structure, presumably due to the 10 Pro residues in its mature 40 amino acids (aa) long periplasmic domain (Fig. 2a, b). It was demonstrated that Rz and Rz1 form a complex through C-terminal interactions [8, 17]. Since this complex spans the periplasm, the two proteins were named as subunits of a spanin complex, with Rz as the prototype i-spanin (IM subunit) and Rz1 as the prototype o-spanin (OM subunit) [18] (Fig. 2a; left). Genes arranged like *RzRz1* were categorized as two-component spanins (2CS), and found to be common in phages of Gram-negative hosts [19], strongly supporting the general role of the spanin complexes in OM disruption and thus, lysis. Furthermore, i-spanin/o-spanin genes could also be found in two other architectures apart from the completely embedded architecture in lambda: overlapped, where the o-spanin gene extends beyond the i-spanin reading frame, and separated, where the genes do not overlap (Fig. 1b). In the same study, it was found that in a few phages, including the paradigm phage T1, the last gene in the lysis cassette (Fig. 1b) encoded a single protein with an OM-lipoprotein signal and a C-terminal TMD (Fig. 2a; right). The T1 gene, *11* was shown to complement the *RzRz1* lysis defect, indicating that gp*11* also played the same role in lysis; i.e., disruption of the OM. Thus, gp*11* was designated as the prototype unimolecular spanin (u-spanin). Primary structure analysis indicated that the periplasmic domain of gp*11* was predicted to be comprised mainly of beta sheets, in contrast to the coiled-coil alpha helices predicted in i-spanins (Fig. 2a, b).

### Molecular function of two-component spanins

Recent studies addressing the molecular mechanism by which the two-component spanin systems function have led to a model for spanin function [10] in which the spanin complex effects removal of the OM barrier by fusing it to the IM (Fig. 2c); thus, spanins mediate a topological solution, rather than a degradative one, for the last step of lysis. The most compelling results supporting the fusion model came from experiments with *E. coli* spheroplasts [10]. Spheroplasts expressing *Rz* were shown to undergo efficient fusion with spheroplasts expressing *imRz1*, a missense allele of *Rz1* in which the lipoprotein OM-localization signal was altered to cause retention in the IM. In contrast, lysis-defective missense alleles of either *Rz* or *Rz1* promoted adhesion of spheroplasts but did not support fusion, indicating that these mutants are blocked at a step after complex formation but before fusion.

A more recent genetic analysis revealed the functional significance of different domains in Rz and Rz1 [17]. Mutations in the coiled-coil domains CC1 and CC2 of Rz rendered the spanin complex non-functional, but the linker domain was shown to act as a flexible spacer, permissive to insertions of Gly-Ser oligopeptide repeats. It was also determined that a proline-rich region (PRR) in Rz1 was mutationally sensitive, with most non-functional mutations mapping to the central penta-proline stretch. Additionally, the N-terminus region of mature Rz1 contained a flexible linker region between the lipoylated Cys and the PRR; Gly-Ser repeat additions to this domain did not affect spanin function. Both coiled-coil domains and PRR domains are known to be a common feature of well-studied membrane fusion motifs in the existing literature [20, 21]. Furthermore, the genetic analysis also provided clues about covariance at different positions and thus potential interaction sites between Rz and Rz1. While characterizing the non-functional spanin mutants, it was observed that complex formation was unaffected for most *Rz* alleles but was defective for *Rz*_*E150G*_ [17]. However, both the lysis and spanin complex-formation defects were suppressed by introducing an R59E change in Rz1, suggesting an interaction between the E150 residue in Rz and the R59 residue of Rz1. In addition, exchanging the charges at these positions, i.e. Rz_E150R_ and Rz1_R59E_ also restored complex formation and lytic function, indicating the interaction occurred through a salt bridge between these residues. To follow up, a suppressor analysis was done for a number of lysis-defective spanin mutants in order to detect more contact points within the Rz-Rz1 hetero-tetrameric complex [22]. Surprisingly, most of the suppressors clustered along the juxtamembrane region of CC1 in Rz and were not allele-specific. These mutations, mostly polar insertions into the hydrophobic core of CC1, were proposed to disrupt the stability of the juxtamembrane region, leading to a conformational state that overcomes the fusion block created by the primary mutation.

Since the first study describing the classification of spanins into two-component spanin and u-spanin systems [19], many new phage genomes have been deposited in the databases [23–25]. Here we present a comprehensive compilation of the spanin genes now identifiable in the public databases and relate features defined by this analysis to the proposed model for spanin function.

## Results and discussion

### Identification of spanins and implications for automated phage annotation

We restricted our primary search to RefSeq genomes of phages of Gram-negative hosts. Spanins have only been recently characterized, compared to the long history of studies on other lysis proteins, and essentially all experimental work has been done with the lambda *Rz* and *Rz1* genes only. Thus, it was not surprising to find that less than 20% of these genomes had both an i-spanin and o-spanin identified; most of these were sequence homologs of the two-component spanin systems from paradigm phages lambda Rz/Rz1, P2 LysB/LysC or T4 PseT.3/PseT.2, due to the over-representation of lambdoid, P2 and T4-like phage genomes in the database. The requirement for an outer membrane lipoprotein signal for both two-component spanins and u-spanin systems suggested that an automated strategy based on identifying genes with such signals could be implemented. However, our initial attempts along this line were frustrated because in most cases, the o-spanin genes in the embedded and overlapped architectures were not annotated as CDSs, reflecting a strong bias against overlapped genes in the most commonly used gene-calling programs like Glimmer [26, 27] or GeneMark [28–30]. The second problem was that many CDSs in phage genomes have misidentified start codons, again probably linked to the bias against overlapped genes in gene-calling programs and the tendency of phage genes to overlap [31]. Since signals necessary to localize proteins to any location in the envelope, including the IM, periplasm, and OM, are always at the N-terminus of a protein, misidentified start codons are extremely problematic for the identification of spanins and, indeed, all lysis proteins. Consequently, we implemented a manual search protocol, shown in Additional file 1: Figure S1, augmented by specialized work-flows constructed at the Center for Phage Technology Galaxy instance [32]. Using this protocol, we interrogated 677 genomes of dsDNA phages of Gram-negative hosts and found 528 two-component spanins and 58 u-spanins, as described in Table 1. The rest of the 91 genomes did not possess any potential spanin candidate genes that met our eligibility requirements for the membrane localization signals. Of the 528 two-component spanin systems, 182, 228 and 118 belonged to the embedded, overlapped and separated architectures, respectively. Additional file 2: Table S1 contains all the coordinates, sequences and other features of the spanins identified in this survey. (Additional file 2: Table S1 serves as the initial basis for a continuously updated SpaninDataBase (SpaninDB) at the Center for Phage Technology website [33]). The results justified our decision for manual annotation. Of the 528 genomes with two-component spanin systems, the CDS for the o-spanin had errors that would preclude automated annotation in 260 cases (196 entirely missing CDSs and 64 CDSs with incorrect start sites) (Table 2 and Additional file 2: Table S1). The problem was less severe with the i-spanins (13 missing, 34 with the incorrect start site) and u-spanins (4 with incorrect start site). These findings suggest that phage-specific algorithms for gene calling are needed before accurate automated analysis of phage genomes can be practical.

**Table 1:** Spanin statistics

Type of spanin system	Number of phages
Embedded 2CS	182
Overlapped 2CS	228
Separated 2CS	118
Unimolecular spanins	58
No spanins found	91
Total	677

**Table 2:** Spanin Annotation problems

Annotation problem	Number of spanin systems
i-spanin not identified	13
o-spanin not identified	196
i-spanin wrong start	34
o-spanin wrong start	64
i-spanin annotated wrong	28
o-spanin annotated wrong	4

As a primary analysis, we first inspected the overall length distribution of spanin complexes, and their secondary structure distribution as predicted by Jpred (Additional file 3: Table S2). It can be expected that since the spanin complex formed by the interaction of the periplasmic domains needs to span the entire periplasm, there would be length restrictions for the number of residues that would be required to physically connect the membranes. However, the potential to adopt various secondary structures and the dependence of periplasmic width on host and environmental conditions, would allow for a varied range of lengths. This was very evident from the periplasmic length profiles of 2CS, both the periplasmic domains put together, ranging from as short as 62 residues for the spanins from phiP27 to 300 residues for the spanins from Marshall (Additional file 3: Table S2). The differences were also noticeable in secondary structure profiles; the first major observation from these predictions was that, in contrast to the unstructured character of the periplasmic domain of Rz1, several embedded spanins were predicted to have a significant structural component. For example, the HK97 o-spanin showed as high as 47% alpha helical character, while the o-spanin from phage HK225 showed as high as 28% beta sheet character. The beta sheet character was more prominently found in the separated o-spanins compared to the overlapped and the embedded architectures. Given the proposed role of the coiled-coil domains in lambda spanin function, we asked if the coiled-coil domains, as predicted by the tool Pepcoil, were conserved among other 2CS (Additional file 4: Table S3). As expected from the secondary structure predictions, the variation of coiled coil character was also very high, with more than 120 of the i-spanins having no coiled-coil predictions at all, and more than 250 i-spanins having one coiled-coil domain. All these differences in structural domain organization of the spanins hint that the eventual goal of spanin function, i.e. membrane fusion of the OM with IM, may be achieved in different ways by different spanin complexes, not necessarily only through coiled-coil domain interactions as proposed in lambda. In conjunction with this theory, we did not observe conserved periplasmic lengths or secondary structure distributions for different phages infecting the same host (Additional file 3: Table S2). Given that the periplasmic width changes depending on the type of the host and growth conditions [34, 35], it cannot be ruled out that spanin complexes of different length might be sequestered to different parts of the periplasm. Extensive structural studies will need to be done to understand the detailed mechanistic differences between spanins of different structures and if any conformational changes occur between free and complex bound spanins.

We also examined the register and position of the o-spanin gene with respect to the i-spanin gene, especially for the embedded and overlapped architectures (Additional file 5: Table S4). All the previously reported o-spanins were found in the + 1 reading frame of their respective i-spanins [19], suggesting that the codon mix available from + 1 frameshifts was required for maintenance of o-spanin function. However, in this survey, we found that the o-spanin occupied the − 1 frame of the i-spanin in 116 cases (Additional file 5: Table S4). Considering the over-representation of homologs of the lambda, T7 and P2 spanins, which are the experimentally tested embedded (lambda and T7) and overlapped (P2) two-component spanin systems and all of them are + 1 architectures, we conclude that there is no significant bias to either the + 1 or − 1 reading frame for o-spanin evolution. Analysis of the relative position of the o-spanin with respect to the i-spanin genes showed that in ~ 70% of the embedded spanins, the o-spanin started at ~ 0.4–0.5 L and ended at 0.9-1 L, where L is the length of the corresponding i-spanin (Additional file 4: Table S3). Combined with our findings from lambda genetics [17], it can be interpreted that the positioning of the embedded o-spanin gene would need to be conserved, as the shared DNA would need to accommodate for the homology of both the CC2 domain of the i-spanin and the C-terminal region of the o-spanin. Since the extreme C-terminus does not seem to be involved in interaction sites for spanin complex formation, the last 0.1 L of the i-spanin is subject to variability.

### Diversity of the two-component spanin systems

To assess the sequence diversity of spanins, we grouped the spanins into families using BLASTCLUST on the CPT Galaxy platform [36], defining families such that every member shared ≥40% identity over ≥40% of the length of the periplasmic sequence with every other member. Focusing on the periplasmic domain avoids the low complexity regions within the IM and OM lipoprotein localization signals, which are necessary for membrane anchoring only and have no functional significance [8, 17]. This approach resulted in the 528 two-component spanins systems being grouped into 157 i-spanin (99 singletons) and 136 o-spanin (65 singletons) families and the 58 u-spanins, into 13 (6 singletons) families. BLASTCLUST uses BLOSUM62, a “deep” scoring matrix that requires long sequence alignments [37, 38]. Thus, all the sequences with a periplasmic domain shorter than 50 aa were combined with the singletons from the BLASTCLUST analysis and manually clustered into new or existing families, as per our definition. This eventually resulted in 143 i-spanin (80 singletons) families, 125 (54 singletons) o-spanin families and 13 u-spanin (6 singletons) families (Tables 3, 4 and 5). A substantial component of the 2CS collection was biased towards homologs of the spanins of the lambdoid phages and T4-like phages. The largest i-spanin family, represented by lambda Rz had 83 members, and the largest o-spanin family represented by T4 PseT.3 had 47 members while a majority of the u-spanins fell under the T1 family with 28 members.

**Table 3:** i-spanin families

Family representative (No. of members)	Members
Embedded	
**Lambda (83)**	Lambda, H-19B, M6, MP1412, PEp14, YuA, Pollock, FSL_SP-058, FSL_SP-076, 1720a-02, Bcep22, BcepMigl, DC1, Gifsy-2, BcepIL02, ES2, Gifsy-1, Fels-1, ST64b, ES18, FSL_SP-016, HK620, SPN3UB, cdtI, mEp460, spn9CC, vB_SosS_Oslo, BP-4795, HK106, HK633, SE1, ST104, VT2-Sakai, YYZ-2008, mEp235, phiSG1, 933 W, ENT39118, Min27, Stx1, Stx2–86, Stx2-II, Stx2-I, Stx2_converting_phage_vB_EcoP_24B, TL-2011c, 21, DE3, Eta, HK629, HK630, pSG3, ST160, ST64T, phi80, SfI, PhiES15, 2851, CUS-3, HK542, HK544, HK75, HK97, Hk022, Sf101, Stx2, c341, epsilon34, mEP234, mEPX1, mEp043_c-1, mEp213, sf6, vB_SemP_Emek, P22, Phi20, Phi75, HK140, HK446, P13374, POCJ13, PS34, mEpX2, phiEt88
**T7 (39)**	T7, vB_EamP-L1, K11, K30, KP32, gh-1, phiPSA2, Berlin, Yep-phi, Yepe2, YpP-G, phi15, 13a, MmP1, PPpW-4, R, T3, T7M, Y, YpP-R, YpP-Y, YpsP-G, phiA1122, phiIBB-PF7A, phiSG-JL2, phiYeO3–12, vB_YenP_AP5, 285P, BA14, CR8, FE44, IME15, Vi06, K1F, PE3–1, EcoDS1, Phi-S1, Kvp1, CR44b
**Bcep176 (15)**	Bcep176, PPpW-3, RSK1, phiPSA1, BcepC6B, KS9, eiAU-183, E1, SPC32H, SPC32N, SPN1S, SPN9TCW, TL-2011b, epsilon15, phiV10
**N4 (12)**	N4, vB_EamP-S6, RG-2014, Bp4, EC1-UPM, JWDelta, ECBP1, pSb-1, vB_EcoP_G7C, JWAlpha, vB_EcoP_PhAPEC5, vB_EcoP_PhAPEC7
Groups of 4	(**B3**, JBD25, JBD18, JBD67), (**KS5,** Smp131, phiRSA1, RSY1)
Groups of 3	(**phiE125**, phi1026b, phi644–2), (**HK225**, ZF40, mEp237), (**LIT1**, vB_PaeP_C2–10_Ab09, Luz7)
Groups of 2	(**BcepMu**, phiE255), (**F10**, vB_PaeP_Tr60_Ab31)
Singletons	phiW-14, Cr30, Xfas53, ECML-117, PY54, N15, BcepB1A, EcP1, S1, F116, phiPLPE, vB_EcoM_ECO1230–10
Overlapped	
**Jersey**^**a**^ **(27)**	Jersey, L13, SETP3, SS3e, wksl3, K1G, K1H, K1ind3, SETP7, vB_SenS-Ent1, vB_SenS-Ent2, vB_SenS-Ent3, vB_SenS_AG11, SE2, SETP13, FSL_SP-101, K1ind1, K1ind2, EK99P-1, EP23, HK578, JL1, SSL-2009a, SO-1, FSL_SP-031_SIS, FSL_SP-038_SIS, FSL_SP-049_SIS
**DMS3 (16)**	DMS3, D3112, F_HA0480sp-Pa1651, JBD24, JBD26, JBD30, JBD5, JBD88A, JD024, LPB1, MP22, MP29, MP38, MP42, MP48, PA1-KOR-2010
**JH2 (15)**	JH2, ECBP2, KBNP1711, NJ01, EC6, FO1a, FSL_SP-010, FSL_SP-012, FSL_SP-107, Felix01, Moogle, Mushroom, PhiEco32, UAB_phi87, WV8
**T5**^**a**^ **(10)**	T5, EPS7, Stitch, SPC35, bV_EcoS_AKFV33, vB_EcoS_FFH1, DT57C, My1, phiR201_SIS, Shivani_SIS
**phiKMV (10)**	phiKMV, LKD16, Luz19, MPK6, MPK7, PT2, PT5, phikF77, vB_Pae-TbilisiM32, phi2
**KS14 (9)**	KS14, ST79, ENT90, Fels-2, RE-2010, phi52237, phiE12–2, phiE202, KL3
**F1 (8)**	F1, Bk, Fz, Pr, R/C, S708, Tb, Wb
**Chi (6)**	Chi, FSL_SP-030, FSL_SP-088, FSL_SP-124, iEPS5, Enc34
Groups of 5	(**P2**, Wphi, fiAA91-ss, L-413C, PsP3), (**phiCbk**, CCrKarma, CcrMagneto, CcrRogue, CcrSwift), (**PR3**, PR4, PR772, PRD1, PR5), (**CP1**, OP1, Xop411, Xp10, phiL7)
Groups of 4	(**Bcep1**, Bcep43, Bcep781, BcepNY3), (**CP8**, NCTC12673, CP30A, CPX), (**KL1**, 73, vB_Pae-Kakheti25, vB_PaeS_SCH_Ab26)
Groups of 3	(**PAK_P1**, vB_PaeM_C2–10_Ab1, JG004), (**9NA**, FSL_SP-062, FSL_SP-069), (**phiEa104**, PhiEa21–4, vB_EamM-M7), (**Era103**, phiEA100, phiEa1H), (**K139**, Kappa, VPUSM_8), (**phiHSIC**, Jenny_12G5, pYD38-B), (**MSW-3**, PEi2, vB_AsaM-56^b^)
Groups of 2	(**phiJL001**, RDJL_Phi_1), (**phiMHaA1**, vB_MhM_1152AP), (**phi92**, phAPEC8), (**SfIV**, SfV), (**Mu**, D108), (**P1**, P7), (**PAP2,** 119X), (**APSE-1**, APSE-2), (**pIS4-A**, pYD38-A), (**Aaphi23**, S1249), (**HP1**, HP2), (**HK639**, mEP390), (**Paz**, Prado^b^), (**phi1402**, phi1422), (**Bf7**, LKA1^b^), (**DFL12phi1**, EE36P1_SOS^b^)^a^
Singletons	OP2,9 g, phiKZ, EL, phiCTX, vB_VpaS_MAR10, 201phi2–1, KPP23, PhiO18P, vB_CsaP_GAP52, KS10, Kpp25, PBC5, Ea35–70, OBP, PAK_P5, PhiPsa374, ENT47670, Xp15, vB_XveM_DIBBI, 7–11, CCrColossus, phiAS7, BcepGomr, phiR8–01, BcepNazgul, Presley, SuMu, phi80–18, vB_CskP_GAP227, RSB3, vB_RleM_PPF1, ESSI-2, Cd1, RSJ2, PY100, RSB1, PM1, AF, vB_EamM-Y2, vB_RleS_L338C, Redjac, phiEcoM-GJ1, vB_RglS_P106B, SSU5, UAB_phi78, phi1M2–2, Salvo
Separated	
**T4 (40)**	T4, Bp7, CC31, JS10, JS98, PG7, ime08, phiR1-RT, vB_YenM_TG1, AR1, ECML-134, PS2, PST, RB14, RB32, RB51, SP18, Shfl2, T4T, e11–2, ime09, pSs-1, phiD1, vB_EcoM-VR20, vB_EcoM-VR7, vB_EcoM_ACG-C40, vB_EcoM_VR25, vB_EcoM_VR26, wV7, RB69, Shf125875, hx01, vB_EcoM_JS09, S16, STML-198, Moon, JSE, Phi1, RB49, vB_EcoM_PhAPEC2
**KP27 (7)**	KP27, Miller, RB43, KP15, Lw1, RB16, vB_CsaM_GAP161
**VP4 (7)**	VP4, ICP3_2007_A^b^, ICP3_2008_A^b^, ICP3_2009_B^b^, ICP3^b^, N4_(Vibrio) ^b^, VP3^b^
**phiAS4 (6)**	phiAS4, 25, 31, 44RR2.8 t, Aes012, Aes508
Groups of 4	(**KVP40**, nt-1, phi-pp2, VH7D), (**4MG**, Av-05, PVP-SE1, vB_CsaM_GAP31), (**D3**, PAJU2, phi297, vB_PaeS_PMG1)
Groups of 3	(**Aeh1**, PX29, phiAS5), (**CP21**, CP220, CPt10), (**rv5**, 2_JES-2013, vB_EcoM-FV3), (**CR3**, CR9, phiTE), (**K1–5**, K1E^b^, SP6^b^)
Groups of 2	(**CC2**, 65), (**PBECO_4**, 121Q)
Singletons	Marshall, B40–8, Pf-WMP3, CR5, phiR1–37, pVp-1, Ac42, vB_CsaM_GAP32, PhiKO2, phi_3, 133, vB_RleM_P10VF, JD001, BcepF1, 1 M3–16, Acj9, AH2, phage_7–7-1, PhiP27, F108, Sano

Representative phages of each family are highlighted in **bold**

^a^indicates family with members from different architectures

^b^indicates members that were manually added to the group after BLAST analysis for short sequences

**Table 4:** o-spanin families

Family representative (No. of members)	Members
Embedded	
**HK97 (32)**	HK97, 2851, CUS-3, HK106, HK140, HK446, HK542, HK544, HK633, HK75, Hk022, P13374, P22, POCJ13, PS34, Phi20, Phi75, ST160, ST64T, Sf101, Stx2, c341, epsilon34, mEP234, mEPX1, mEp043_c-1, mEp213, mEp235, mEpX2, phi80, sf6, vB_SemP_Emek
**Lambda (28)**	Lambda, PhiES15, phiSG1, phiEt88, pSG3, SfI, ES18, Fels-1, Gifsy-2, ST64b, 1720a-02, 933 W, BP-4795, H-19B, Min27, Stx1, Stx2–86, Stx2-II, Stx2-I, Stx2_converting_phage_vB_EcoP_24B, TL-2011c, VT2-Sakai, YYZ-2008, cdtI, 21, DE3, HK629, HK630
**Bcep176 (15)**	Bcep176, RSK1, SPC32H, SPC32N, SPN1S, SPN9TCW, TL-2011b, epsilon15, phiV10, BcepC6B, KS9, eiAU-183, PPpW-3, E1, phiPSA1
**HK620 (14)**	HK620, PEp14, FSL_SP-058, FSL_SP-076, Pollock, Gifsy-1, FSL_SP-016, SE1, SPN3UB, ST104, mEp460, spn9CC, vB_SOSS_Oslo, ENT39118
**K11 (14)**	K11, K30, KP32, MmP1^b^, vB_EamP-L1^b^, IME15^b^, phiSG-JL2^b^, phiYeO3–12^b^, vB_YenP_AP5^b^, EcoDS1^b^, K1F^b^, PE3–1^b^, CR44b^b^, CR8^b^)
**N4 (12)**	N4, vB_EamP-S6, Bp4, EC1-UPM, JWAlpha, JWDelta, ECBP1, RG-2014, pSb-1, vB_EcoP_G7C, vB_EcoP_PhAPEC5, vB_EcoP_PhAPEC7
**T7 (11)**	T7, 13a, R, T3, T7M, Vi06, Y, YpP-R, YpP-Y, YpsP-G, phiA1122
**Kvp1 (8)**	285P, BA14, Berlin, FE44, Kvp1, Yep-phi, Yepe2, YpP-G
Groups of 4	(**B3**, JBD18, JBD67, JBD25), (**KS5**, Smp131, RSY1, phiRSA1), (**phi15**, PPpW-4, Phi-S1, phiIBB-PF7A)
Groups of 3	(**LIT1**, Luz7, vB_PaeP_C2–10_Ab09), (**phiE125**, phi1026b, phi644–2), (**M6**, MP1412, YuA), (**HK225**, ZF40, mEp237), (**BcepIL02**, BcepMigl, ES2^b^)
Groups of 2	(**BcepMu**, phiE255), (**Bcep22**, DC1), (**F10**, vB_PaeP_Tr60_Ab31), (**phiPSA2**, gh-1)
Singletons	vB_EcoM_ECO1230–10, N15, Ecp1, ECML-117, PY54, BcepB1A, F116, S1, phiW-14, Cr30, Xfas53, Eta, phiPLPE
Overlapped	
**Jersey**^**a**^ **(27)**	Jersey, FSL_SP-101, K1G, K1H, K1ind1, K1ind2, K1ind3, L13, SE2, SETP13, SETP3, SETP7, SS3e, vB_SenS-Ent1, vB_SenS-Ent2, vB_SenS-Ent3, vB_SenS_AG11, wksl3, FSL_SP-031_SOS, FSL_SP-038_SOS, FSL_SP-049_SOS, EK99P-1, EP23, HK578, JL1, SO-1, SSL-2009a
**DMS3 (16)**	DMS3, D3112, F_HA0480sp-Pa1651, JBD24, JBD26, JBD30, JBD5, JBD88A, JD024, LPB1, MP22, MP29, MP38, MP42, MP48, PA1-KOR-2010
**P2 (15)**	P2, ENT90, ST79, Fels-2, L-413C, PsP3, RE-2010, Wphi, fiAA91-ss, KL3, KS14, phi52237, phiE12–2, phiE202, phiCTX
**JH2 (11)**	JH2, EC6, FO1a, FSL_SP-010, FSL_SP-012, FSL_SP-107, Felix01, Moogle, Mushroom, UAB_phi87, WV8
**T5**^**a**^ **(10)**	T5, My1, DT57C, EPS7, SPC35, Stitch, bV_EcoS_AKFV33, vB_EcoS_FFH1, Shivani_SOS, phiR201_SOS
**BcepNazgul**^**a**^ **(10)**	BcepNazgul, 73, vB_Pae-Kakheti25, vB_PaeS_SCH_Ab26, KL1, HK639, mEP390, PY100, AF, AH2_SOS
**phiKMV (9)**	phiKMV, LKD16, Luz19, MPK6, MPK7, PT2, PT5, phikF77, vB_Pae-TbilisiM32
**Chi (8)**	Chi, SSU5, Enc34, FSL_SP-030, FSL_SP-088, FSL_SP-124, iEPS5, Redjac
**F1 (8)**	F1, Bk, Fz, Pr, R/C, S708, Tb, Wb
**Mu (7)**	Mu, D108, ENT47670, SfIV, SfV, APSE-1, APSE-2
**CP1 (7)**	CP1, phiL7, OP1, Xop411, Xp10, Prado, Paz
**phiCbk (6)**	phiCbk, CCrKarma, CcrMagneto, CcrSwift, CCrColossus, CcrRogue
**PR3 (5)**	PR3(tecti), PR4(tecti), PR5(tecti), PR772(tecti), PRD1(tecti)
Groups of 4	(**Bcep1**, Bcep43, Bcep781, BcepNY3), (**CP8**, CP30A, CPX, NCTC12673), (**PAK_P1**, JG004, PAK_P5, vB_PaeM_C2–10_Ab1), (**9NA**, ESSI-2, FSL_SP-062, FSL_SP-069), (**NJ01**, ECBP2, KBNP1711, PhiEco32), (**MSW-3**, PEi2, vB_AsaM-56, JD001_SOS)^a^
Groups of 3	(**K139**, Kappa, VPUSM_8), (**phiEa104**, PhiEa21–4, vB_EamM-M7), (**Era103**, phiEA100, phiEa1H), (**phiHSIC**, Jenny_12G5, pYD38-B)
Groups of 2	(**KPP23,** RDJL_Phi_1), (**phi1402**, phi1422), (**phi92**, phAPEC8), (**phiAS7**, phiR8–01), (**phiMHaA1**, vB_MhM_1152AP), (**HP1**, HP2), (**P1**, P7), (**Aaphi23**, S1249), (**pIS4-A**, pYD38-A), (**PAP2**, 119X), (**PM1**, phiEcoM-GJ1), (**RSB1**, Cd1^b^) (**Bf7**, phi2, LKA1^b^), (**SuMu**, vB_EamM-Y2^b^), (**DFL12phi1**, EE36P1_SOS)^a^, (**Salvo**, Sano_SOS)^a^
Singletons	Ea35–70, OBP, EL, Xp15, phiKZ, 201phi2–1, phiJL001, 9 g, vB_XveM_DIBBI, KS10, PBC5, Kpp25, OP2, phi80–18, vB_RleM_PPF1, BcepGomr, vB_CsaP_GAP52, 7–11, vB_CskP_GAP227, PhiO18P, Presley, RSB3, vB_VpaS_MAR10, RSJ2, vB_RleS_L338C, vB_RglS_P106B, PhiPsa374, phi1M2–2
Separated	
**T4 (47)**	T4, phiAS5, Ac42, Moon, CC2, CC31, PG7, 65, AR1, ECML-134, JSE, PS2, PST, Phi1, RB14, RB32, RB49, RB51, Shfl2, T4T, e11–2, ime09, pSs-1, phiD1, vB_EcoM_ACG-C40, wV7, PX29, phiR1-RT, vB_EcoM-VR7, vB_EcoM_VR25, vB_YenM_TG1, 133, Aeh1, Bp7, JS10, JS98, RB69, S16, SP18, STML-198, Shf125875, hx01, ime08, vB_EcoM-VR20, vB_EcoM_JS09, vB_EcoM_PhAPEC2, vB_EcoM_VR26
**KP27 (7)**	KP27, Lw1, Miller, RB16, RB43, KP15, vB_CsaM_GAP161
**rv5 (7)**	rv5, 2_JES-2013, Av-05, vB_EcoM-FV3, 4MG, PVP-SE1, vB_CsaM_GAP31
**VP4 (7)**	VP4, ICP3_2007_A, ICP3_2008_A, ICP3_2009_B, ICP3, N4_(Vibrio), VP3
**phiAS4 (6)**	phiAS4, 25, 31, 44RR2.8 t, Aes012, Aes508
Groups of 4	(**KVP40**, nt-1, VH7D, phi-pp2), (**D3**, PAJU2, phi297, vB_PaeS_PMG1), (**K1–5**, K1E, SP6, UAB_phi78_OOS)^a^
Groups of 3	(**CR3**, CR9, phiTE), (**PBECO_4**, 121Q, vB_CsaM_GAP32), (**CP21**, CP220, CPt10)
**pVp-1** (2)	pVp-1, phi_3
Singletons	CR5, B40–8, phiR1–37, Marshall, vB_RleM_P10VF, Acj9, 1 M3–16, phage_7–7-1, PhiKO2, BcepF1, Pf-WMP3, PhiP27, F108

Representative phages of each family are highlighted in **bold**

^a^indicates family with members from different architectures

^b^indicates members that were manually added to the group after BLAST analysis for short sequences

**Table 5:** u-spanin families

Family representative (No. of members)	Members
**T1 (28)**	Limezero, J8–65, 1513, bV_EcoS_AHP42, bV_EcoS_AHS24, bV_EcoS_AKS96, e4-1c, phiEB49, phiJLA23, phiKP26, vB_EcoS_Rogue1, pSf-1, JK06, KP36, RTP, vB_EcoS_ACG-M12, Shfl1, T1, pSf-2, LIMELIGHT, F20, FSL_SP-126, Stevie, TLS, F19, KP34, NTUH-K2044-K1–1, phiKDA1
**PB1 (11)**	PB1, 14–1, F8, SPM-1, JG024, KPP12, LBL3, NH4, SN, lma2, vB_PaeM_PAO1Ab27
**phi13:2 (4)**	phi13:2, phi18:3, phi19:3, phi47:1
**phi12:1 (3)**	phi12:1, phi17:1, phi18:1
Groups of 2	(**phi10:1**, phi19:1), (**VpV262**, CW02), (**SIO1**, P12053L)
Singletons	RAP44, 11b, CHOED, FCL-2, 9A, ICP2_2013_A_Haiti

Representative phages of each family are highlighted in **bold**

The difference between the number of i-spanin and o-spanin families (143 versus 125, respectively) was intriguing. This could be partly attributed to the difference in lengths and aa composition of the spanin counterparts, considering the way BLOSUM62 similarity scores are calculated [38]. Long sequence alignments are easier to achieve in the i-spanin components than the o-spanin components; the i-spanin component is longer than the corresponding o-spanin component in 489 of the 528 two-component spanins described in this study. The difference in number and composition of i-spanin and o-spanin families also reflects the distinct evolutionary paths of spanin components, independent of each other. For example, in the case of lambda spanins, the Rz family had 83 members, whereas the respective o-spanin counterparts were distributed into 7 different families, most of them spread across three families HK97 (32 members), lambda (28 members) and HK620 (14 members). This essentially means that the 83 i-spanins could be arranged into alignments with statistically significant scores, enough to cluster them into a family according to our criteria, whereas the alignments of their o-spanin counterparts did not lead to scores sufficient for all of them to be included into one single family. Instead, the alignments of the 83 o-spanins could be divided into different groups, where the scores within each of the individual group allowed them to be clustered into a family. This suggested that the members within these o-spanin families were more closely related to each other than members from other families, in terms of evolutionary distance. This observation was surprising, as it indicated that these o-spanin sequences diverged more than i-spanin sequences, even though they were totally embedded within the latter. This means that the segment of the i-spanin gene that contains the coding sequence for the periplasmic domain of the o-spanin in the + 1 frame would have to be more permissive for changes, while the rest of the gene was relatively conserved. This interpretation was consistent with the findings from our recent genetic analysis of the lambda spanins [17]. The DNA encoding the periplasmic domain of Rz1 is shared with the genetically flexible linker region in Rz. Notably, even though the genetic study was done on a synthetic separated pair of Rz-Rz1, the mutational clusters of one gene corresponded to the mutationally silent region in the other, highlighting the differential evolutionary pressures on the embedded spanins.

Interestingly, when we repeated the clustering with 100% sequence identity over 100% sequence length (full length, including the signal sequences), we found that there were 46 sets of 2CS, spread across the three different genetic architectures, and 3 sets of u-spanins accounting for 113 and 6 sequences respectively, each set having entirely identical sequences (Table 6). Remarkably, a few of the identical spanins came from phages that infected different hosts, which reinforces the mosaic nature of phages [39]. For example, Enterobacteria phages T3 and T7M share identical embedded spanins with Yersinia phages R, YpP-Y and YpP-R. The unexpectedly high frequency of identical spanins was probably a byproduct of our strategy of using a BLAST-DB of existing spanins to hunt for new spanins (Additional file 1: Figure S1). Nonetheless, this strategy helped us identify spanins in new phage genomes, even if identical to already existing spanins, where they had not been identified before or even annotated. Even with the presence of a high number of identical spanins, we still observed 80 i-spanin and 54 o-spanin singleton classes, or ~ 55% and 43% of the total number of families of i-spanins and o-spanins respectively having only one member, highlighting the extreme diversity of this functional class of lysis proteins.

**Table 6:** Identical spanin families

Family representative (No. of identical members)	Members
Embedded	
**T3 (7)**	T3, R, T7M, Y, YpP-R, YpP-Y, phiA1122
Groups of 4	(**SPC32H**, SPC32N, SPN1S, SPN9TCW), (**933 W**, Stx1, Stx2-II, Stx2_converting_phage_vB_EcoP_24B), (**Lambda**, DE3, HK629, HK630)
**P22 (3)**	P22, Phi20, Phi75
Groups of 2	(**phiE125,** phi1026b), (**SE1**, ST104), (**Stx2-I**, Stx2–86), (**ST160**, ST64T), (**Stx2**, 2851), (**HK75**, HK544), (**mEp213**, mEp043_c-1), (**Yepe2**, YpP-G), (**BA14**, FE44)
Overlapped	
**F1 (8)**	F1, Bk, Fz, Pr, R-C, S708, Tb, Wb,
**JBD5 (5)**	JBD5, F_HA0480sp-Pa1651, MP29, MP48, PA1-KOR-2010,
Groups of 3	(**DMS3**, JBD30, MP42), (**MP22**, JBD88A, MP38), (**vB_SenS-Ent1**, vB_SenS-Ent2, vB_SenS-Ent3), (**Era103**, phiEA100, phiEa1H), (**Felix01**, FO1a, UAB_phi87), (**phiKMV**, PT5, vB_Pae-TbilisiM32)
Groups of 2	(**K139**, Kappa), (**phiE12–2**, phi52237), (**phiMHaA1**, vB_MhM_1152AP), (**Bcep43**, Bcep781), (**P1**, P7), (**SS3e**, wksl3), (**phiEa104**, PhiEa21–4), (**FSL_SP-062**, FSL_SP-069), (**K1ind1,** K1ind2), (**pIS4-A**, pYD38-A), (**FSL_SP-010**, FSL_SP-012), (**Aaphi23**, S1249), (**Chi**, FSL_SP-030)
Separated	
**ICP3 (5)**	ICP3, ICP3_2007_A, ICP3_2008_A, ICP3_2009_B, N4_(Vibrio),
**Shfl2 (4)**	Shfl2, PST, ime09, pSs-1,
**FSL_SP-031 (3)**	FSL_SP-031, FSL_SP-038, FSL_SP-049,
Groups of 2	(**PBECO_4**, 121Q), (**AR1**, wV7), (**ECML-134**, vB_EcoM_ACG-C40), (**Miller**, RB43), (**KP27**, KP15), (**Aes012**, 25), (**phiAS4**, Aes508), (**VP4**, VP3)
Unimolecular	
Groups of 2	(**bV_EcoS_AHS24**, vB_EcoS_Rogue1), (**F8**, SPM-1), (**phi13:2**, phi19:3)

Phages representing their identical partners in the non-identical set are highlighted in **bold** (See methods)

### Conserved functional domains and covariance

As a follow up on the genetic analysis of the lambda spanins [17], we investigated the alignments of the different spanin family members to see if the mutationally sensitive regions and the functional domain organization was conserved. We hoped to visualize covariance amongst residues that could potentially be involved in primary and secondary site interactions, important for different stages of the spanin operation. We performed multiple sequence alignments of the i-spanin and o-spanin sequences of all families, based on both i-spanin and o-spanin clustering. However, in this report we limit ourselves to discuss the alignments of the one family from each genetic architecture that gave us the most significant insights into conserved regions.

**Fig. 3:**
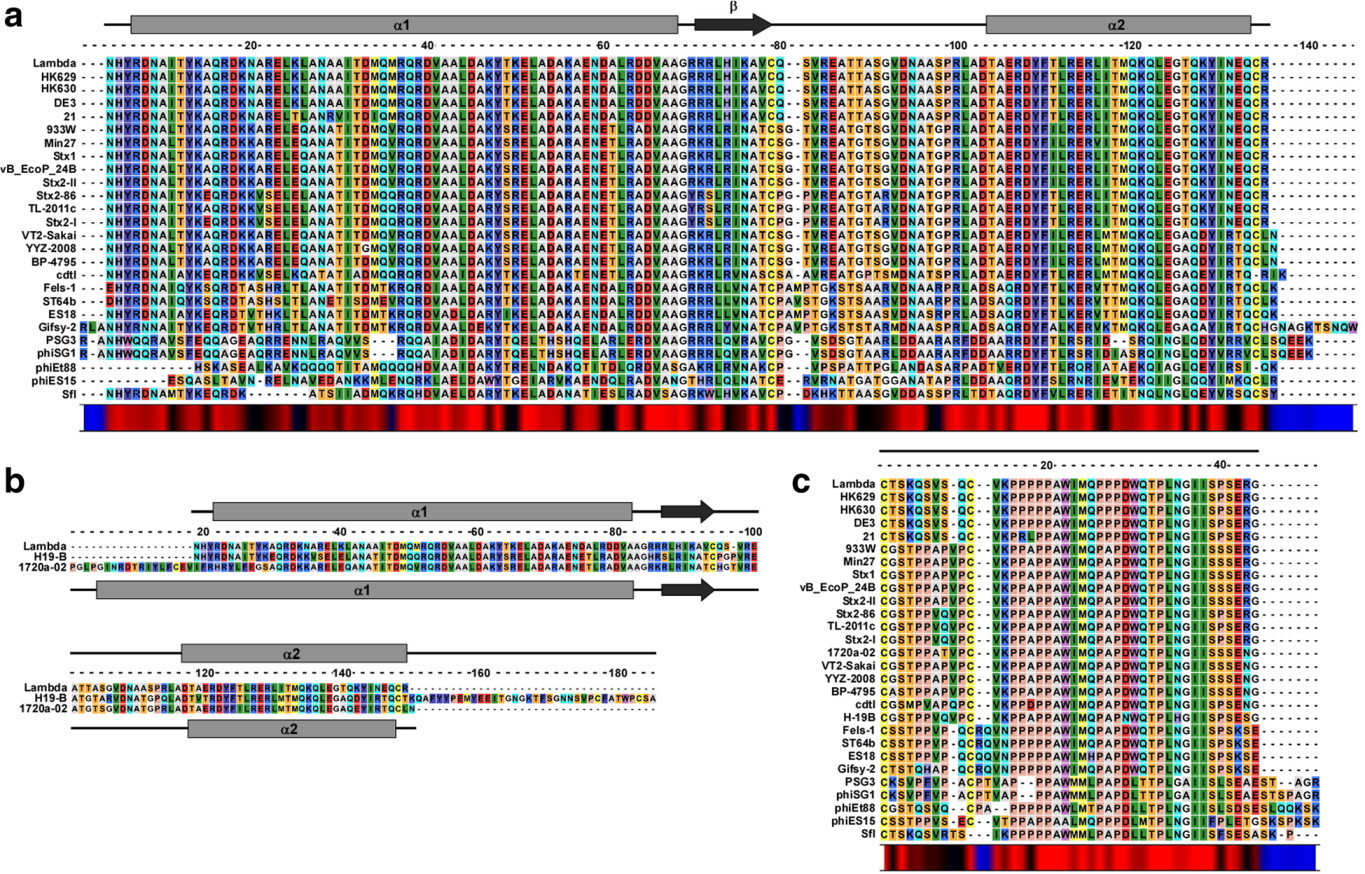
Sequence alignments of lambda spanin families. **a** and **c** show alignments of i-spanin and o-spanin sequences (periplasmic domains only) of the lambda family respectively. The alignments of i-spanin sequences from 1720a-02 and H-19B with respect to the lambda i-spanin are shown separately in (**b**) as their i-spanin sequences were significantly longer compared to other members. Secondary structure predictions for respective spanins from lambda are shown above each alignment. The secondary structure predictions for i-spanins from H19B and 1720a-02 are shown above and below respectively, in the additional alignment in (**b**). Grey rectangles indicate alpha helices and black arrows indicate beta sheets. Conservation at different positions is shown below with colors (Red indicates maximum conservation and blue indicates least conservation). A scale indicating the relative position of the residues and the approximate size of various domains is shown above the alignments

For the embedded architecture, we analyzed the alignments and secondary structure organization of sequences from 26 members of the lambda o-spanin family (Table 4) and their i-spanin counterparts. The alignments revealed highly conserved regions in both spanin components that were consistent with the mutationally sensitive positions identified in the experimental genetic analysis [17]. The regions of alignment of i-spanins corresponding to mutationally sensitive regions of α1 (between residues R59 and A88) and α2 of lambda Rz (between residues D126 and Y147), were conserved while the linker region (between residues Q100 and S115) showed variability in composition, in accordance with the proposed flexibility from the genetic studies (Fig. 3a). The periplasmic domains from i-spanins of 1720a-02 and H19-B were remarkably longer than the other members. Upon alignment, we noticed that this was a result of addition of long aa stretches at the extreme N-terminus for 1720a-02 (19 residues) and C-terminus for H19-B (37 residues) (Fig. 3b). Secondary structure predictions showed that the N-terminal additional segment in 1720a-02 is predominantly alpha-helical albeit with no coiled-coil character, whereas the C-terminal additional segment in H19-B is mostly unstructured. We hypothesize that the segments are probably dispensable and not involved in interactions necessary for spanin function. Based on our findings with lambda spanins [17], we proposed the N-terminal region of 1720a-02 might act as a long helical linker connecting the α1 to the IM and the C-terminal domain in H19-B might act as flexible loop allowing the distal end of α2 to interact with the o-spanin. Upon inspecting the o-spanin alignments (Fig. 3b), we found the penta-proline stretch in lambda Rz1 was conserved in all the other o-spanin members, except for P34, which was also the only position in that stretch where no lysis-defective mutations were found [17], underscoring the permissive nature of the position. In addition, the linker region between the N-terminal lipoylation Cys and the penta-proline stretch was relatively variable, gaining more prolines which would add to the unstructured character of this region. This agreed with our genetic analysis [17] which showed that the linker region was flexible and allowed for the addition of Gly-Ser repeats. The o-spanin sequences from PSG3, phiSG1, phiEt88, phiES15 and SfI all had longer, and variable C-terminal ends compared to the other members. However, these additional segments might not be relevant to spanin function as the lambda genetics showed the extreme C-terminus, though involved in interactions with the i-spanin counterpart, is dispensable [17].

The lambda spanin family alignments also revealed that the Cys residues at position 29 in the o-spanin and 99, 152 in the i-spanin are unchanged in almost all members, except for cdtI, phiEt88 and SfI. cdtI and phiEt88 lack the C152 in the i-spanin, whereas SfI lacks the C29 in the o-spanin. However, we expect these spanins to still be functional as the cysteine substitutional analysis of lambda Rz-Rz1 showed that the presence of just one Cys in either of these positions was necessary and sufficient for spanin function [18]. It was notable that the C99 is a constant feature in all these i-spanins despite being dispensable for spanin function, indicating there might be some advantage in terms of structural stability conferred by the intermolecular disulfide bond at that position. Furthermore, the alignments also supplemented the findings of our suppressor analysis [22]. For examples, secondary site suppressors Rz_Y33C_ and Rz_A47S_ were isolated against the lysis-defective Rz_V61A_ allele, while Rz_D38G_ was isolated against Rz_L64H_. We examined these specific positions in the alignments and found that the i-spanins from both PSG3 and phiSG1, which contained an Ile instead of Val at position 61 and Ile instead of Leu at position 64, also had substitutions at positions 33 (Y33➔F), 38 (D38➔G) and 47 (A47➔L). This strongly suggested covariance at these positions, but it is not clear if these sites are involved in interaction, as there seems to be no allele specificity. The covariance between Rz1_R59_ and Rz_E150_ was also quite evident, adding to our salt bridge interaction findings. Using the alignments, we could identify other covariance patterns, such as Rz1_W46➔L_- Rz_E142➔N_ and Rz1_V26➔A_ -Rz1_P56➔S_. However, it remains to be tested if these residues are involved in interactions relevant for spanin function.

**Fig. 4:**
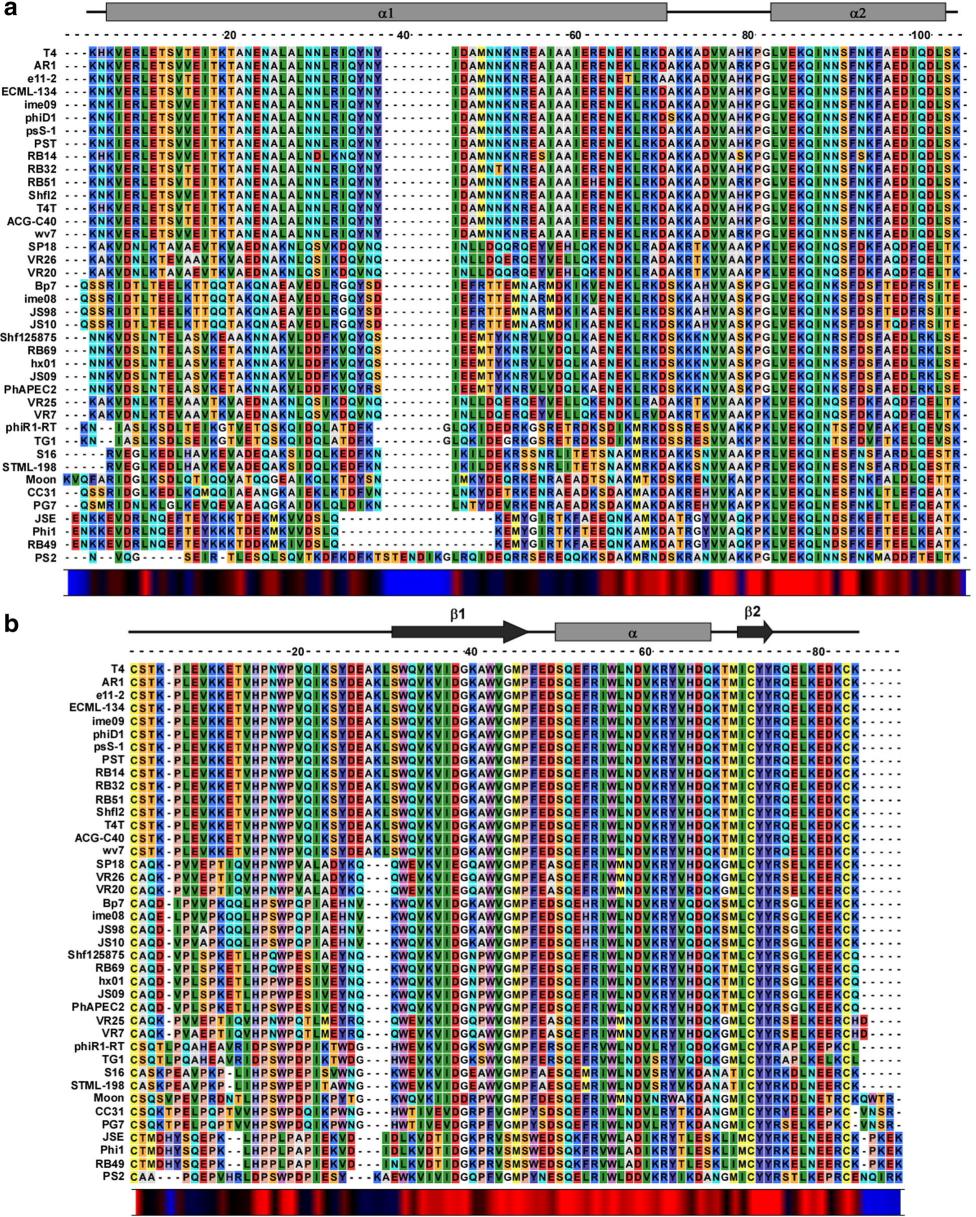
Sequence alignments of T4 spanin families. **a** and **b** show alignments of i-spanin and o-spanin sequences (periplasmic domains only) of the T4 family respectively. Secondary structure predictions for respective spanins from T4 are shown above each alignment. Grey rectangles indicate alpha helices and black arrows indicate beta sheets. Conservation at different positions is shown below with colors (Red indicates maximum conservation and blue indicates least conservation). A scale indicating the relative position of the residues and the approximate size of various domains is shown above the alignments

For the separated architecture, we analyzed the alignments of sequences from 40 members of the T4 i-spanin family and their o-spanin counterparts, shown in Fig. 4a, b respectively. Secondary structure predictions for the T4 spanins are significantly different from the lambda spanins. The periplasmic domain of T4 i-spanin, PseT.3 essentially consists of two alpha helices, designated as α1 and α2 (Fig. 4a), connected by a very short linker region. However, unlike lambda Rz, only α1 has the propensity to form coiled-coils. In comparison to the unstructured lambda Rz1, the T4 o-spanin PseT.2 contains a β1-α-β2 domain organization, a flexible linker connecting the OM to the central beta sheet domain, followed by an alpha helix motif, and a very short beta sheet, while the extreme C-terminus has no predicted structural content. The alignments showed significantly conserved regions as well secondary structure organization in both spanin components. Like lambda, the T4 o-spanin family seemed to have a highly variable linker region between the N-terminal Cys linking to the OM and the central beta sheet domain (Fig. 4b). The β1-α-β2 stretch ranging from W47 to R90 was greatly conserved. Unlike the lambda o-spanin family, the T4 o-spanin family lacks any proline rich region or proline stretches. Still, the Pro residues at positions 21, 31, 34 and 61 were conserved in almost all the members, suggesting these residues might be involved in the fusion activity. Moreover, both the Cys residues at positions 87 and 98, were unchanged in all members, emphasizing the role of the intra- and intermolecular disulfide bonds at these positions for spanin function. On the other hand, the i-spanin alignments showed a conserved α1 and α2 domain organization, even though the N-terminus of α1 (connecting to the IM) region was extremely variable in composition. Even with the extreme variation in α1, α2 was conserved across all the members, starting around L96 up to the C-terminal end, implying this domain could be involved in potential interaction with the conserved regions of the o-spanins. Upon inspection of these alignments for intra- and intergenic covariance, we observed that the a negatively charged residue at position 92 in the o-spanin was almost always associated with the presence of a K117 in the i-spanin, suggesting these two residues could be involved in polar interactions, probably through a salt bridge, at some stage of the spanin pathway.

**Fig. 5:**
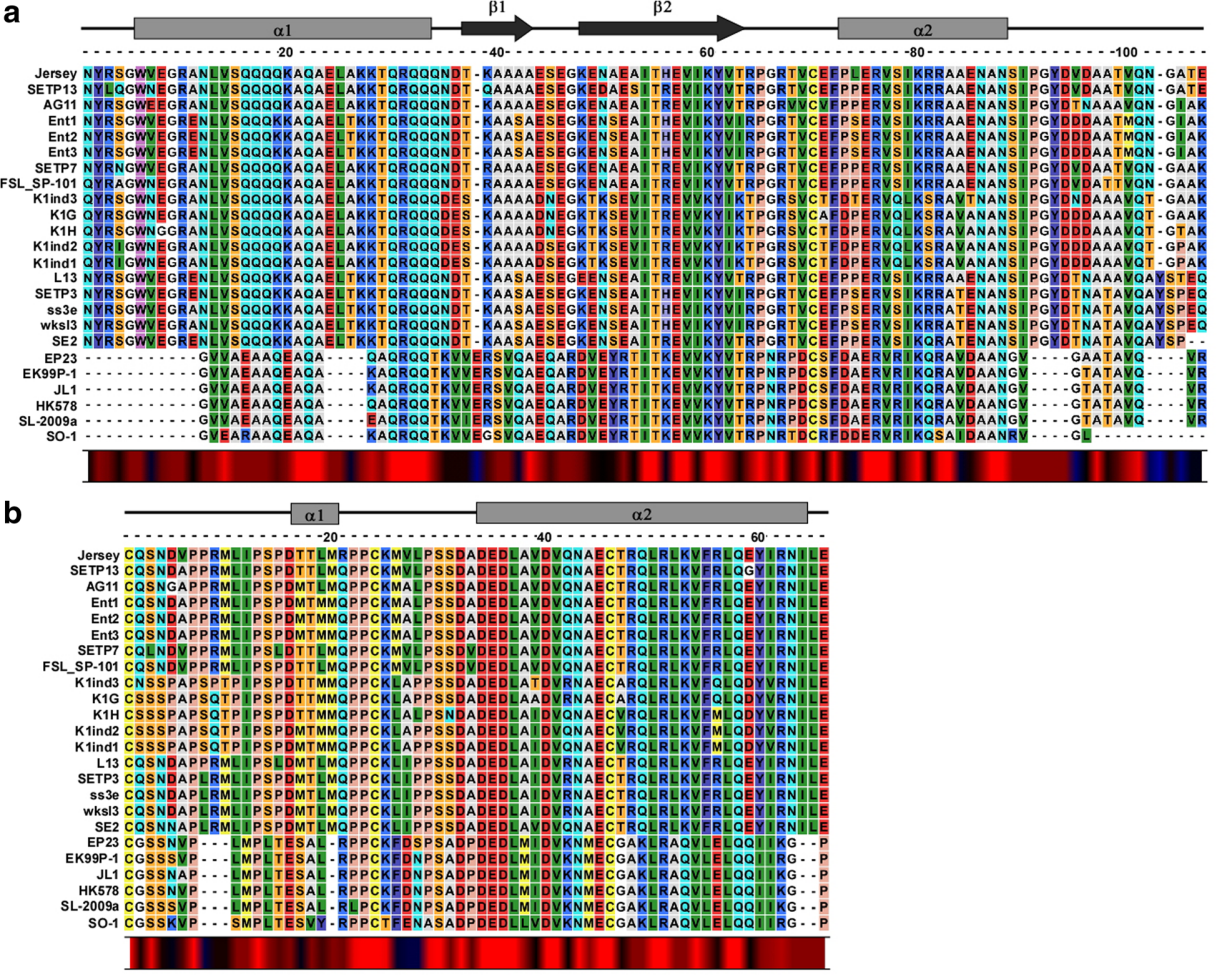
Sequence alignments of Jersey spanin families. **a** and **b** show alignments of i-spanin and o-spanin sequences (periplasmic domains only) of the Jersey family respectively. Secondary structure predictions for respective spanins from Jersey are shown above each alignment. Grey rectangles indicate alpha helices and black arrows indicate beta sheets. Conservation at different positions is shown below with colors (Red indicates maximum conservation and blue indicates least conservation). A scale indicating the relative position of the residues and the approximate size of various domains is shown above the alignments

For the overlapped architecture, we analyzed the alignments of sequences from 24 members of the Jersey i-spanin family (Table 3; we omitted the sequences from FSL_SP-031, − 038, and − 049 as they belonged to the separated genetic architecture. Alignments including these sequences are discussed in the next section.) and their o-spanin counterparts as shown in Fig. 5a, b. The alignments showed fairly conserved sequences and secondary structure organization spread across the periplasmic domains of both the spanins. The Jersey i-spanin family could be broadly classified into two subfamilies, based on the length of the periplasmic domains and the predicted secondary structure profiles. The first subfamily (Jersey to SE2 in Fig. 5a) had a secondary structure organization of two alpha helices connected by a linker region. But unlike the unstructured linker as seen in lambda, the linker region of these i-spanins had 2 predicted beta sheets, the first instance of i-spanins showing significant beta sheet secondary structure character. The second subfamily (EP23 to SO-1 in Fig. 5a), shorter in length, had a similar two alpha helix domain organization, with only one beta sheet motif predicted in the linker region. The extreme C-terminal end of both the subfamilies was highly variable as well as unstructured, signifying the α2 region, not the extreme C-terminus, was probably involved in interaction with the i-spanin. Interestingly, the first subfamily also contained two polyQ stretches. PolyQ repeats are known to facilitate and stabilize coiled-coil interactions [40], and could potentially affect spanin function in this case by strengthening homotypic interactions between adjacent spanin complexes. All the o-spanins had a conserved 2 alpha helical prediction (denoted as α1 and α2 in Fig. 5b) connected by a flexible linker, in addition to the flexible linker region connecting the N-terminal Cys to the central short helix. The position and composition of α1 varied in different members, while α2 was relatively more conserved, again suggesting involvement in interactions with i-spanin. Both Cys at positions 45 and 67 remained constant, hinting at the potential role of intermolecular disulfide bonds in spanin function here. Prolines were also conserved in different positions of both the unstructured linker regions.

### Evolution of the two-component spanin genetic architecture: caught in the act

In a recent study of phages isolated against *Xylella* and *Xanthomonas*, we described a new phage type, represented by the phage Nazgul [41], consisting of 7 phages; Nazgul, Redjac, Enc34, Chi and AH2, Sano and Salvo. While characterizing these phages, we identified and grouped the spanin sequences into 2 families, one consisting of the phages Sano and Salvo, and the others including phages Nazgul, Redjac, Enc34, Chi and AH2. Interestingly, while Salvo had its two-component spanins in an overlapped architecture, the spanin gene pair in Sano was separated. Similarly, the spanins of AH2 were separated, while the respective homologs from other members of the family, Nazgul, Redjac, Enc34 and Chi, were all overlapped. Not only was this the first report showing spanin gene families with members across different architectures, but also the alignments of these spanin sequences from different architectures hinted at the evolution process of the two-component spanin architecture. While almost all the two-component spanin families were restricted to a single architectural class based on our grouping strategy, we found three i-spanin families and seven o-spanin families that had members from both overlapped and separated architectures. For further analysis, we chose the three families represented by DFL12phi1, T5, and Jersey, where both the i- and o-spanin families had members from the overlapped and separated architectures and hoped to predict the potential evolution pathways that could transition from one genetic architecture to the other.

**Fig. 6:**
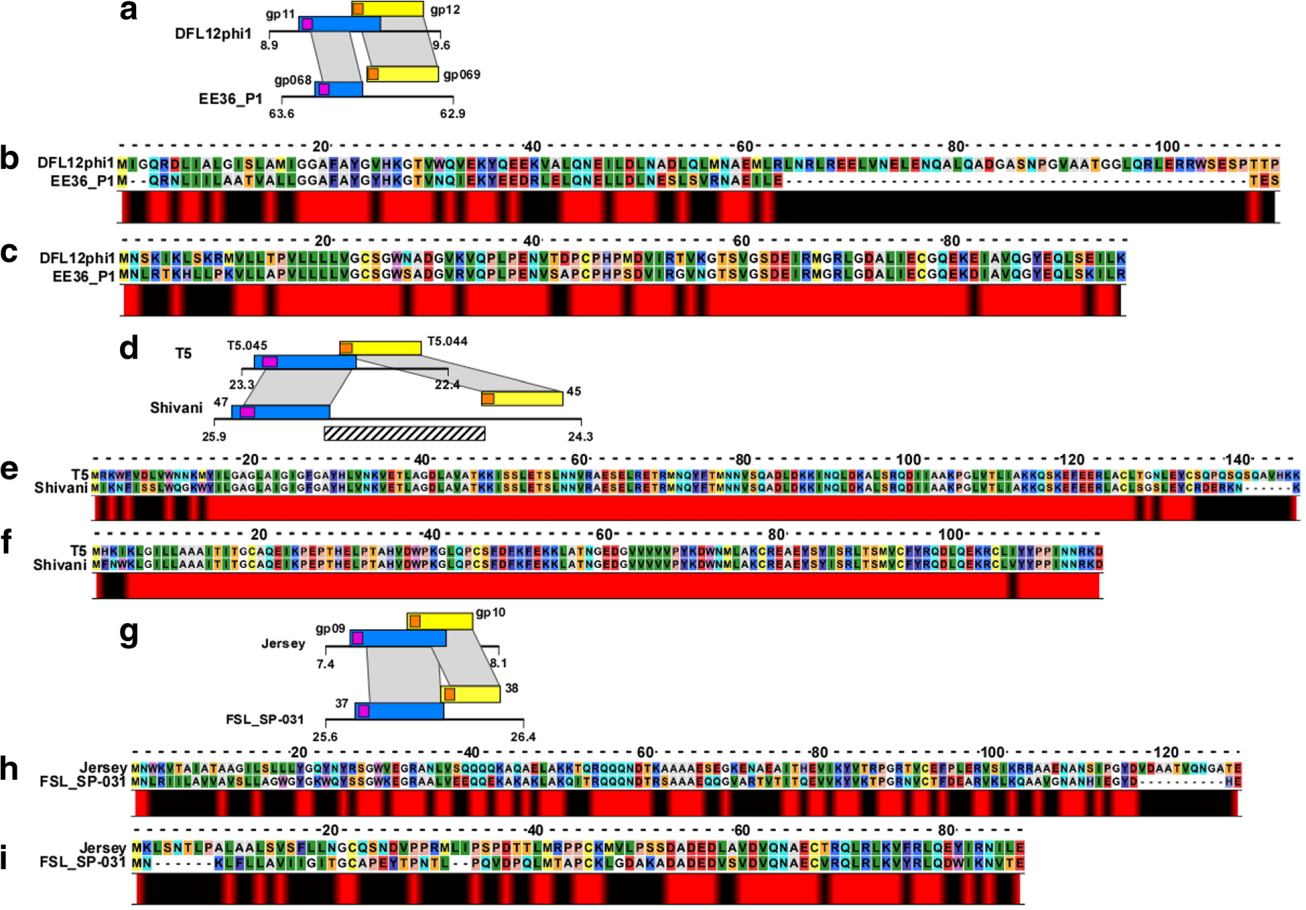
Sequence alignments of spanin sharing homology across different architectures. **a**, **d**, **g** are pictorial representations of the homologous regions of DLF12 and EE36P1, T5 and Shivani. and Jersey and FSL_SP-031 respectively. **b**, **c** show sequence alignments of periplasmic domains of i-spanin and o-spanin of DLF12 and EE36P1 respectively, (**e**, **f**) show sequence alignments of periplasmic domains of i-spanin and o-spanin of T5 and Shivani respectively and (**h**, **i**) show sequence alignments of periplasmic domains of i-spanin and o-spanin of Jersey and FSL_SP-031 respectively. We chose to include the full-length sequences in the alignments as the shared DNA region codes for the signal sequence of the o-spanin. Blue and yellow rectangles represent i-spanin and o-spanin respectively, whereas the pink and orange rectangles represent the approximate position of the N-terminal TMD sequence in the i-spanin and the N-terminal lipoylation signal sequence in the o-spanin respectively. Grey shaded region indicates the region of sequence similarity between homologs. Gene names are in bold, all genes are drawn to scale, and their position in the genome in terms of kilobases is denoted by the numbers below the line. Conservation at different positions is shown below with colors (Red indicates maximum conservation and blue indicates least conservation). A scale indicating the relative position of the residues and the approximate size of various domains is shown above the alignments

The first family consists of the overlapped spanins from DFL12phi1 and the separated spanins from EE36P1. The spanins in DFL12phi share 116 bp, whereas the spanins in EE36P1 are separated by 17 bp (Fig. 6a). Upon comparing the alignments and secondary structure predictions (Fig. 6b, c), we observed that the i-spanin from EE36P1 has a conserved N-terminal alpha helix α1 domain like the DFL12phi i-spanin, but the entire C-terminal stretch including the second predicted alpha helix α2 is missing. The o-spanins are conserved all throughout the periplasmic domain, maintaining the secondary structure domains as well. The T5 family, consisted of nine members, of which seven were of the overlapped genetic architecture; EPS7, Stitch, SPC35, bV_EcoS_AKFV33, vB_EcoS_FFH1, DT57C and T5 and the other two, phiR201 and Shivani, were of the separated genetic architecture. To simplify the analysis, we chose T5 to represent the overlapped class and aligned its two-component spanins with the sequences from Shivani. Upon examination, we found that in Shivani and phiR201 the spanin pair are separated by ~ 700 bp that includes a gene encoding for a putative GIY_YIG homing endonuclease, whereas the T5 spanins overlap by 70 bp (Fig. 6d). The i-spanins are almost identical across the periplasmic domain, except for the extreme C-terminal end, which extends into the o-spanin gene (Fig. 6e). The o-spanins are almost identical across the entire length, except for the few residues of the lipoylation signal sequence (Fig. 6f). Naturally, the spanins from both the phages have very similar secondary structure predictions. Lastly, the Jersey family consisted of 28 members, of which 25 fell under the overlapped gene arrangement and the 3 are in the separated class (Tables 3, and 4). To simplify our alignments, we chose Jersey and FSL_SP-031 to represent the overlapped and separated classes, respectively. The spanins in Jersey overlap by 155 bp, while the spanin pair in FSL_SP-031 have only 8 bp of overlap and are thus separated according to our criteria (Fig. 6g). The i-spanins share homology across the entire length, except for the N-terminal TMD and the extreme C-terminal end (Fig. 6h). The o-spanins share little to no homology at the N-terminus, including the lipoylation signal sequences, but are highly similar from the central to C-terminal end (Fig. 6h).

Each of these three cases of families sharing members from different architecture seems to be hinting at a different mode of evolution between architectures. Comparing the reading frames in the shared region of DFL12phi1 to the corresponding region in EE36P1, it can be hypothesized that an in-del mutation or a premature stop codon at the C-terminal of i-spanin resulted in the separated architecture in EE36P1. However, it remains to be tested if the short i-spanin of EE36P1 is still functional without the extended C-terminus, as in DFL12phi1. In the case of T5 and Shivani, transition may have happened from an overlapped to separated architecture or vice versa depending on if the homing endonuclease gene was inserted or deleted at the C-terminal end of the i-spanin. In either case, the insertion or deletion would need to happen in a manner such that the newly generated N-terminus of the o-spanin gene still encoded the lipoylation signal sequence. The most parsimonious interpretation of the findings from Jersey and FSL_SP-031 alignments is that the spanin genes started with a separated spanin architecture, and the transition from separated to overlapped architecture involved a duplication of the entire spanin locus, followed by deletion event. The fact that average length of both the spanin genes put together is not more than 250 residues, i.e. ~ 0.75 kb, implies that the duplication is very probable. That would allow the phage to manipulate one copy of the spanin pair, through deletions and other mutations, until the duplicated copy is not only functional but also shorter. Once both the copies are functional, the longer version can be deleted. The variability in the extreme C-terminus of the i-spanin allows for the transition where the o-spanin is integrated in the + 1 reading frame. Our suppressor analysis [22] using a synthetic separated pair of Rz-Rz1 illustrated that the embedded architecture did not confer any advantage in terms of functional efficiency. Thus, it seems that the only advantage gained is that of increased coding capacity.

In all the three cases, the o-spanins retain their sequence homology throughout the predicted periplasmic domains of the separated and overlapped proteins, but the i-spanins diverged in the C-terminal region encoded by the DNA containing the actual overlap. This suggests that the interaction between the i-spanin and o-spanin in both the overlapped and separated architectures does not depend on the extreme C terminus of the o-spanin, as was demonstrated for the lambda Rz-Rz1 proteins of the embedded architecture. There were no families that had members from the embedded architecture combined with either overlapped or separated architecture. Nevertheless, a similar argument can be put forth that a duplication followed by deletion process could have generated the embedded gene architecture from the separated arrangement. A more complex pathway for arriving at the embedded architecture could be envisioned where a mutation in the stop codon of the i-spanin gene allows it to extend until a new stop beyond the end of the downstream o-spanin gene. This would need to be followed by internal deletions within the non-overlapping region in the i-spanin to restore the overall length. The intermediate i-spanin in this pathway could easily be fully functional, since the addition of a few random residues at the C-terminus of the i-spanin would not necessarily affect the ability to interact with the o-spanin. Moreover, the unusual amino acid content of o-spanins (e.g., lambda Rz1 has 10 Pro residues in its mature 40 aa length) and lack of required secondary structure could facilitate the generation of new i-spanin segments in the + 1 or − 1 reading frame relative to the o-spanin. In support of this notion, Rancurel et al. [42] have shown that genes arising as out of frame sequences embedded within a gene encoding a structured protein, tend to be enriched in disorder-promoting residues (residues that tend to stay unstructured; e.g. Pro, Glu, Ser, Lys [43]). Considering our findings here, the most parsimonious interpretation would be that the embedded and overlapped architectures evolved in parallel, unrelated pathways from primordial separated genes.

## Intermolecular disulfide linkages as a universal feature of two-component spanin systems

**Fig. 7:**
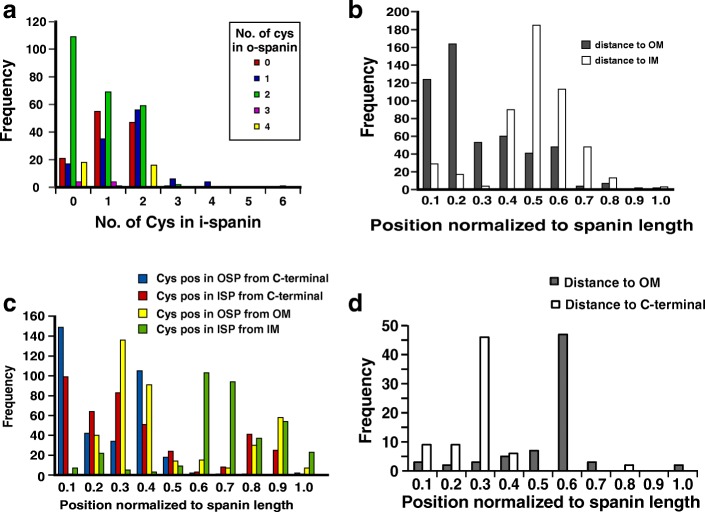
Statistical analysis of cysteines in spanins. **a**: Histogram showing the distribution of periplasmic cysteines in o-spanins w.r.t number of cysteines in their i-spanin counterparts. X-axis indicates the number of cysteines in i-spanins while the Y-axis indicates the number of spanin systems with that combination of periplasmic cysteines in i- and o-spanins. Red, blue, green, purple and yellow indicate the populations with 0,1,2,3, or 4 cysteines in the o-spanin. **b**: Histogram showing the distance distribution of periplasmic cysteines in spanins closest to the OM (grey bars) and IM (white bars). **c**: Histogram showing the distance distribution of periplasmic cysteines in spanins; closest cysteine from the OM in o-spanin (yellow bars), closest cysteine from the IM in i-spanin (green bars), closest cysteine from the C-terminal interaction site in o-spanin (blue bars) and closest cysteine from the C-terminal interaction site in i-spanin (red bars). **d**: Histogram showing the distance distribution of periplasmic cysteines in spanin from the OM (grey bars) and C-terminal interaction site (white bars) in spanin systems with only one periplasmic cysteine

In the context of spanins, the work of Berry et al. [18] on the lambda Rz and Rz1 proteins showed that the mature complex had 3 homotypic intermolecular disulfide bonds, mediated by the two cysteines at position 99 and 152 in Rz and the sole cysteine at position 29 in Rz1. Genetic analysis revealed that lytic function was preserved as long as at least one of the intermolecular bonds at Rz C152 or Rz1 C29 was retained. The formation of these intermolecular disulfide bonds was dependent on the host Dsb system, and a model for this dependence has been described [18]. We wanted to determine if periplasmic intermolecular disulfide bonds could be a common requirement for all two-component spanin function and thus inspected the spanin sequences for cysteines. Indeed, we found that, 507 of the 528 two-component spanins we had identified had at least one cysteine in either the i-spanin or the o-spanin component (Additional file 6: Table S5a). The maximum number of total periplasmic cysteines for two-component spanins was 8, with a maximum of 6 for the i-spanin and 4 for the o-spanin (Tables 7, 8 and 9). The variation in frequency of cysteines in o-spanins with respect to the number of cysteines in i-spanins is shown in (Fig. 7a). The most common combination seemed to be 0 cysteines in i-spanin and 2 cysteines in o-spanin, like PseT.3 and PseT.2 from phage T4. As evident from the correlation graph, as the number of cysteines in the i-spanin increases from 0 to 2, the need for an o-spanin to possess a cysteine or more decreases.

**Table 7:** Total cysteine statistics

No. of total cysteines	No. of spanins
0	21
1	72
2	191
3	130
4	87
5	8
6	16
7	0
8	1
Total	526

**Table 8:** Cysteines in i-spanin statistics

No. of cysteines in i-spanin	No, of spanins
0	169
1	164
2	179
3	9
4	4
5	0
6	1
Total	526

**Table 9:** Cysteines in o-spanin statistics

No. of cysteines in o-spanin	No. of spanins
0	124
1	118
2	240
3	9
4	35
Total	526

The result from Berry et al. [18] that either C29 in Rz1 or C152 in Rz, but not C99 in Rz, was required for the function of the lambda spanin complex indicated that the position of the cysteine, and thus, the covalent disulfide linkage, was important. We hypothesized that the cysteine either needs to be proximal to the interaction site of the C-terminal ends of the spanin components or proximal to the OM attachment site of the spanin complex. We extracted the entire periplasmic sequences of the spanin complexes and analyzed the position of the cysteine closest to the IM and OM attachment sites (Additional file 6: Table S5b and Fig. 7b). The probability of finding a cysteine close to the OM was higher compared to finding one close to the IM. Then we inspected individual spanin components and analyzed the position of the cysteine closest to the C-terminal interaction site and membrane attachment site (IM for the i-spanin, OM for the o-spanin) (Additional file 6: Table S5c, d and Fig. 7c). The cysteines in both the i-spanin and o-spanin components were closer to the C-terminal interaction site, mostly within 40% of the sequence length of the individual spanins. The proximal cysteine distance to OM in the o-spanin was much less than the distance of the proximal i-spanin cysteine to the IM, with the former peaking ~ 30% of sequence length of the o-spanin, while the latter peaked ~ 60–70% of sequence length of the i-spanin. To understand whether the proximity of the cysteine to the OM or the C-terminal interaction site was important for function, we analyzed the subset of spanins with only one cysteine and examined the position of the lone cysteine across the periplasmic domain length (Additional file 6: Table S5e and Fig. 7d). In all cases, the lone cysteine was closer to the heterotypic interface than the OM, suggesting that a cysteine closer to the interaction site stabilizes the interaction between the spanin complexes.

**Fig. 8:**
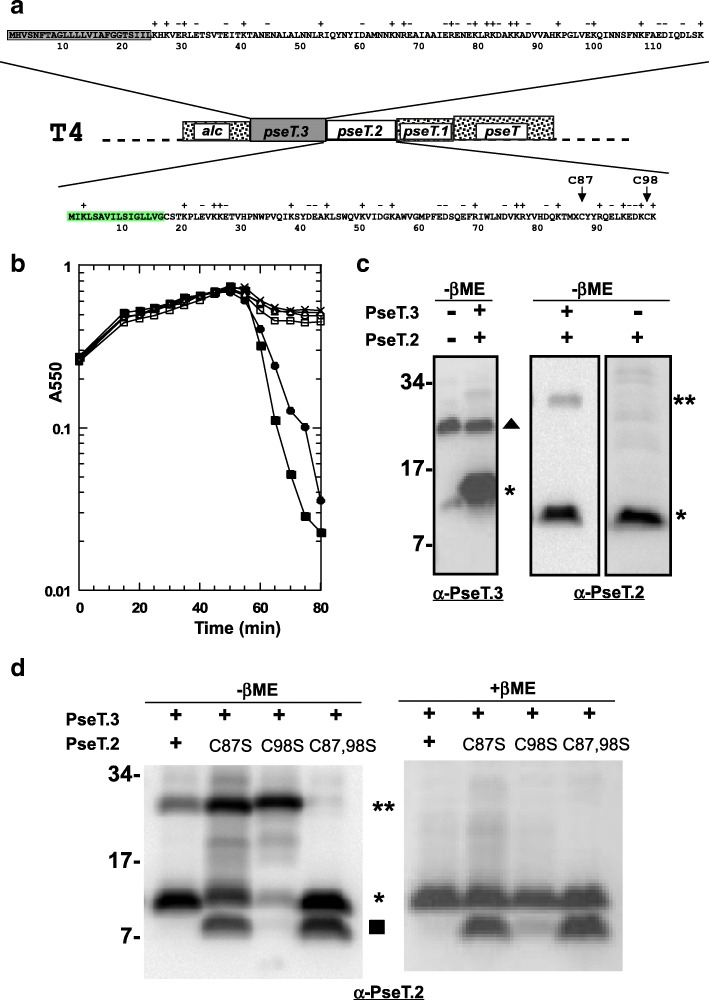
**a**: Primary structure analysis of the separated spanins *pseT.3* (i-spanin) and *pseT.2* (o-spanin) from T4. Unlike the traditional lysis cassette as in lambda, these genes are not located near the T4 holin (*t*) and endolysin (*e*). The inset shows the predicted primary structures of PseT.3 and PseT.2 with their TMD and signal sequence highlighted in gray and green, respectively. *pseT.3* (black) encodes a 117 aa i-spanin and *pseT.2* encodes an 83aa o-spanin. The T4 o-spanin has a predicted helix at the C-terminus and is comparatively larger than the o-spanin in lambda which lacks any predicted or detected helical structure. The position of predicted coiled coil structures in the i-spanin are shown by open rectangles. The two periplasmic cysteines in PseT.2, at positions 87 and 98 are shown by arrows. **b**: Lysis profiles of T4 spanin cysteine mutants. MC4100 (*λ900Rz*_*am*_*Rz1*_*am*_) lysogens grown in LB supplemented with 10 mM MgCl_2_, carrying the following plasmids, were induced at time = 0 and growth was monitored at A550: pRE (−X-); p*RzRz1* (−■-); p*pseT.3pseT.2* (−●-); p*pseT.3pseT.2*_*C87S*_ (−□-); p*pseT.3pseT.2*_*C98S*_ (−Δ-); p*pseT.3pseT.2*_*C87,982S*_ (−○-). **c**, **d**: Western blot analysis of T4 spanin cysteine mutants: TCA precipitates from induced MC4100 (*λ900Rz*_*am*_*Rz1*_*am*_) lysogens carrying the indicated allele were prepared and analyzed in the absence or presence of β-mercaptoethanol as indicated above the gel. For each analysis, the spanin antibody used is indicated at the bottom of each panel. The location of monomeric and dimeric species of PseT.3 and PseT.2 are indicated by (single asterisk) and (double asterisk). Additionally, putative degradation products are indicated by square on the right of each blot. Filled triangles indicate a background band. The alleles are indicated above each lane. Molecular markers in kDa are indicated to the left

To supplement our findings on whether the periplasmic cysteine requirement is general for all two-component spanins, we decided to test if cysteines were required in systems other than lambda. The paradigm phage T4 not only has a spanin pair with a different architecture (separated) but also a different arrangement of periplasmic cysteines; the i-spanin PseT.3 has no cysteines whereas the o-spanin PseT.2 has two cysteines at position 87 and 98 (Fig. 8a). First, to confirm the functionality of the T4 spanins, we tested the ability of *pseT.2* and *pseT.3* to complement the spanin lysis defect in a *λSRRz*_*am*_*Rz1*_*am*_ background and found that they were functionally equivalent to *Rz-Rz1* (Fig. 8b). However, both single and double Cys to Ser substitution alleles of *pseT.2* generated, were found to be lysis-defective (Fig. 8b), indicating that, unlike the case in the lambda spanins, both the cysteines in the o-spanin were essential for lysis. When analyzed by western blotting under non-reducing conditions, the T4 i-spanin PseT.3 did not form an SDS-stable dimer in vivo*,* as expected due to the lack of periplasmic cysteine residues (Fig. 8c). Unexpectedly, only 30% of the o-spanin PseT.2 was found to be in dimers sensitive to reducing agent (Fig. 8c, d). This PseT.2 disulfide-bonded dimer species was detected only in the presence of PseT.3, suggesting a pathway in which PseT.2-PseT.3 complexes form first, with the former carrying the two Cys residues in an intramolecular disulfide linkage, followed by an isomerization resulting in the formation of two intermolecular linkages between the cognate Cys residues (Fig. 8d). The formation of this doubly-linked complex is required, since the proteins with single intermolecular linkages are non-lytic, despite an enhanced level in both cases (Fig. 8d, lanes 2 and 3). Indeed, the cysteines are conserved in those positions throughout the members of the T4 o-spanin family. Thus, the T4 system, although conforming to the general rule of requiring at least one homotypic intermolecular disulfide bond, is more constrained than the lambda system, in terms of requiring two such linkages.

In both cases examined so far, lambda and Τ4, it is intriguing that the requirement for covalent linkages through the disulfide bonds involves homotypic i-spanin or o-spanin dimers. A requirement for heterotypic i-spanin/o-spanin linkages would not have been surprising, in that it would have provided a covalent bridge between the IM and OM, as is indeed the case for the u-spanins. However, this would require the presence of at least one cysteine in both the spanin components, which is not the case as found in this study. A possible explanation for this observation is that the spanin complexes are subjected to robust forces at the interaction site orthogonal to the axis of the complex, rather than along it. In this scenario, a covalent link via an intermolecular disulfide bond near the junction of spanin interaction in either of the spanin components is necessary to counter these extreme forces. It is possible the intermolecular linkage also stabilizes the collapsed conformation hypothesized for spanin function (Fig. 2c).

Approximately 4% of the two-component spanins were found to lack periplasmic cysteines entirely. While sequencing errors can potentially result in false-negatives (spanins with no cysteines) for this analysis, the number seems too high to be attributed to just sequencing quality. However, none of these phages have been tested for lysis morphology, and thus it would be premature to speculate on how the lack of the covalent linkage is compensated either intragenically or by another as yet unidentified lysis factor.

## Diversity of the unimolecular spanins

**Fig. 9:**
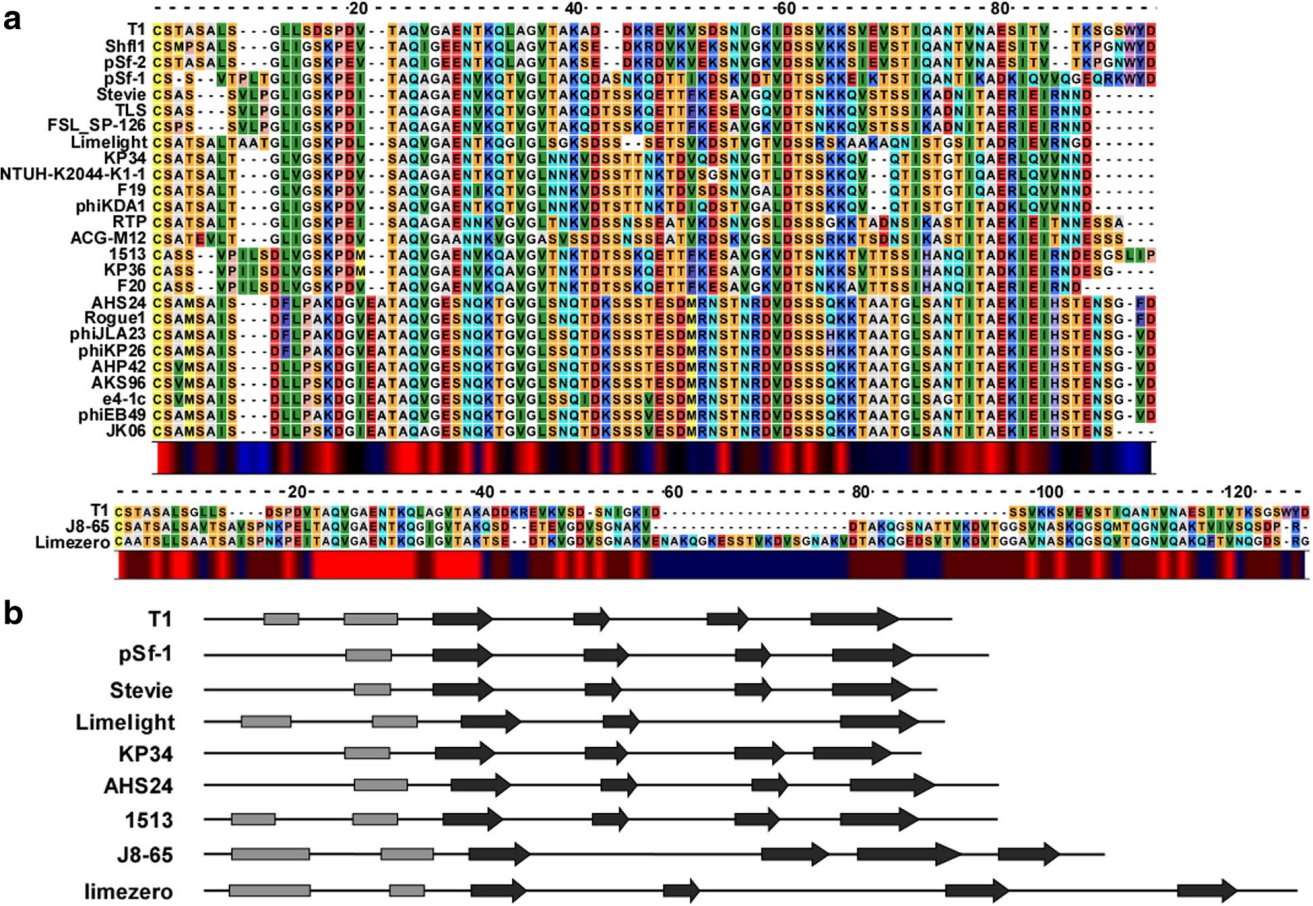
**a**: Sequence alignment of the T1gp*11* family. Shown here is the alignment of the periplasmic domain of members of the T1gp*11* family. The sequences are labelled with the respective phage names on the left. Conservation at different positions is shown below with colors (Red indicates maximum conservation and blue indicates least conservation). A scale indicating the relative position of the residues and the approximate size of various domains is shown above the alignments. **b**: Secondary structure analysis of the periplasmic domain of T1gp*11* family. Shown here is a secondary structure distribution of representative members within the T1gp*11* family with varying predictions. Grey rectangles indicate alpha helices and black arrows indicate beta sheets and are drawn to scale, according to their respective lengths

Using the same strategy as used to group two-component spanins into families, the 58 u-spanins were grouped into 13 families, of which 6 were singletons (Table 5). Of these 58 u-spanins, there were 3 pairs of identical u-spanin sequences that were identified by grouping sequences that were 100% identical over 100% sequence length (Table 6). The family represented by T1gp*11* was the largest with 28 members. Upon aligning the sequences of the members of the T1 family, we noticed various residues spread throughout the periplasmic domain that seem to be involved in predicted secondary structures, were conserved across all members (Fig. 9a). Indeed, analysis of predicted secondary structure revealed that members of the gp*11* family, although different in lengths, maintain similar beta sheet secondary structure distribution (Fig. 9b). A short alpha helical stretch followed by 4 or more beta sheet elements, connected through flexible linkers, was the dominant pattern of the periplasmic domain of all the family members. Even J8–65 and Limezero, which were unusually long and had the least sequence similarity to other members from the gp*11* family, preserved the secondary structure pattern. The next largest family consisted of 11 members, all from *Pseudomonas* phages represented by PB1. Notably, all of these u-spanins possessed an unusual lipobox motif, AWAC (see next section).

All the members of the T1gp*11* u-spanin family, except for J8–65 and Limezero, were part of lysis cassettes encoding a pinholin and SAR-endolysin, initially suggesting that this type of lysis system probably favored the u-spanin over a two-component spanin system. However, when we analyzed the lysis cassettes of all the phages encoding u-spanins, of the 46 genomes where we could identify the endolysin, 29 were SAR endolysins and 17 were canonical endolysins (Additional file 2: Table S1). Thus, it can be concluded that the u-spanins can function with either of the two types of holin-endolysin systems. In contrast to the ubiquitous presence of cysteines in the two-component spanins, none of the 58 u-spanins contained a periplasmic cysteine residue. Thus, we conclude that the constraint in the two-component spanin systems that requires a covalent homodimer linkage near the heterotypic interface does not apply to u-spanin systems.

The diversity and fundamentally different primary and secondary structure characteristics strongly indicate that u-spanins evolved independently of the two-component spanins. One evolutionary path could be that the u-spanins could have originated from an o-spanin, in which a mutation in the stop codon resulting in extension of the C-terminal domain, gaining a C-terminal TMD. However, none of the u-spanins have detectable similarity to any o-spanin, so this scenario remains speculative. There is not yet any physiological or biochemical data addressing how the u-spanins function. Nevertheless, given that both the spanin complex and the u-spanin physically connect the IM and OM, a model for u-spanin function can be proposed on lines, similar to the previously proposed model for two-component spanin function [10]. The most parsimonious idea is that u-spanins also effect OM disruption by causing IM-OM fusion (Fig. 2c), albeit through a collapsing conformational change involving beta-sheets rather than coiled-coil helical domains. The differences in secondary structure content and periplasmic cysteines, suggest that the u-spanins, even though achieving the same end result as the two-component spanins, function in a fundamentally different way. Proteins with very different structural composition accomplishing the same result has been seen before in viral membrane fusion proteins [20, 21]. For instance, class I viral membrane fusion proteins predominantly contain alpha helical domains, whereas the class II viral membrane fusion proteins are rich in beta sheets. The two-component spanins can be considered analogous to the class I viral membrane fusion proteins, while the unimolecular spanins can be compared to the class II viral membrane fusion proteins [20].

While genetic analysis of the *Rz/Rz1* [17, 22] has given us clues about the intermediate steps of the membrane fusion process by two-component spanins, mechanistic details of how u-spanins achieve OM disruption are lacking due to the limited experimental work. A thorough genetic analysis of T1gp*11* to find non-functional mutants blocked at various steps in the lytic pathway is needed. Moreover, assuming that the u-spanin pathway terminates in an analogous OM-IM fusion event analogous to that proposed for the two-component spanins, a spheroplast fusion assay similar to the one used for two-component spanins needs to be designed to exploit the mutant collection. Biochemical experiments testing the post translational processing pathway of u-spanins and potential oligomerization of u-spanin molecules at different stages of the proposed model can also be informative.

### AWAC, a newly identified lipobox

**Fig. 10:**
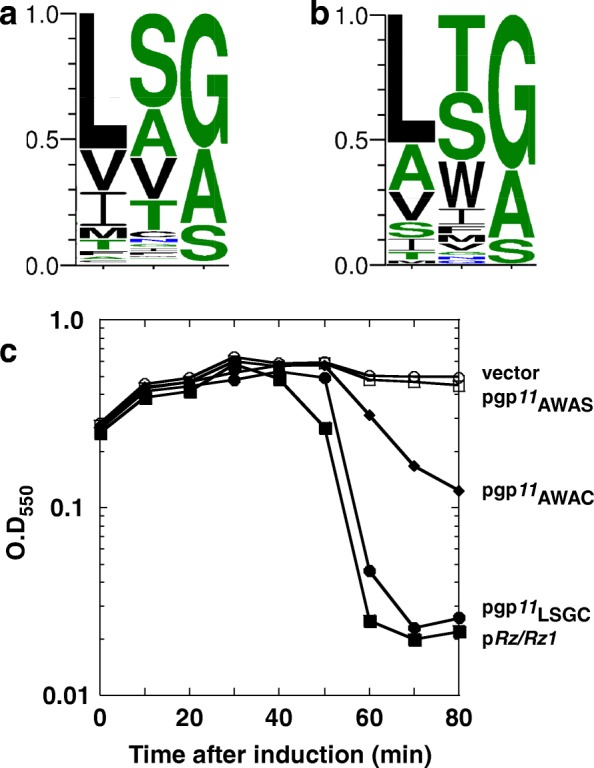
**a**, **b**: Consensus lipobox sequences for o-spanins(a) and u-spanins(b). The three residues signify the three residues immediately before the lipoylation cysteine. Lipobox sequences were collected from respective spanins and plotted using Webb Logo 3.3. *n* = 528 for a) o-spanins and 58 for b) u-spanins. **c**. Lysis profiles of gp*11* lipobox variants: MC4100 (*λ900Rz*_*am*_*Rz1*_*am*_) lysogens grown in LB supplemented with 10 mM MgCl2, carrying the following plasmids, were induced for lysis at time = 0 and growth was monitored as A550: pRE vector (−○-); p*RzRz1* (−●-); pgp*11* (−■-); pgp*11*_*AWAC*_ (−♦-); pgp*11*_*AWAS*_ (−□-)

One of the byproducts of this survey of o-spanins was the identification of novel lipobox sequences. The post-translational processing and sorting of lipoproteins from their nascent form to the mature form has been very well studied [44]. The current consensus lipobox sequence, determined by analyzing the signal sequences of existing lipoproteins over the years is [LVI][ASTVI][GAS][C] [45, 46]. The consensus sequence for the lipoboxes of o-spanins and u-spanins identified in this work, i.e., the 3 residues before the putative lipoylation cysteine, were plotted using Webb Logo 3.3 (Fig. 10a, b) [47]. While most o-spanins still followed the consensus for the lipobox, there were a number of deviations, including AWAC, LNGC, FVGC etc. (Additional file 6: Table S5). We decided to test one of these unusual lipoboxes, AWAC, as no lipoproteins have been experimentally confirmed to have such a lipobox sequence. Moreover, this lipobox was found only in a family of u-spanins, represented by PB1 (Table 5), all of which were associated with the pinholin-SAR endolysin systems. To test whether AWAC is really a lipobox and thus if the u-spanins identified could be functional, we constructed a *gp11* construct where the wild type lipobox LSGC was substituted with AWAC. *gp11*_AWAC_ was able to complement the *λRzRz1* lysis defect (Fig. 10c), demonstrating that AWAC is a valid lipobox motif.

## Spanin-less phages: Is there another route to outer membrane disruption?

In approximately 13% of the genomes analyzed, neither a two-component nor a u-spanin system could be identified (Table 10). There are multiple reasons that would explain our inability to identify spanins in these genomes, the first being sequencing errors. For example, the *Escherichia* phage ADB-2 contains a gene, B508_00385, that encodes a protein highly similar to the u-spanin T1gp*11* (75% identical and e-value of 2e-48), but does not have an N-terminal lipoylation signal sequence. The fact that this gene is adjacent to the predicted holin (B508_00375) and endolysin (B508_00380) genes, presumably as part of a lysis cassette, adds to the speculation that it could be a u-spanin, but the reading frame was not detected because of a sequencing error. A more common problem affecting the search for spanin candidates is the quality of annotation of many phage genomes. All the three spanin genes have N-terminal localization signals, either OM lipoprotein or IM N-terminal TMD; recognition of such signals depends absolutely on having the correct initiation codon. However, gene-calling programs often fail at this because of a bias against CDS overlap [48, 49], which is very common in phage genomes. In phage genomes where the lysis genes were clustered, or where BLAST hits to known spanins were found, it was possible for us to manually correct the gene starts. There were 311 cases in which a spanin was either annotated with an incorrect start codon (34 i-spanins, 64 o-spanins and 4 u-spanins) or not annotated at all (13 i-spanins and 196 o-spanins). However, in cases of T4-like phage genomes, lysis genes are not clustered, making it difficult to ascertain whether gene-calling errors resulted in missing spanin genes.

**Table 10:** Spanin-less phages

Phage Name	Host
AB3, Petty, Abp1, YMC-13-01-C62, vB_AbaM_Acibel004, ZZ1	*Acinetobacter*
pAh6-C	*Aeromonas*
vB_AmaP_AD45-P1	*Alteromonas*
A-4 L	*Anabaena*
B124–14	*Bacteroides*
BIP-1, BMP-1, Bpp-1	*Bordatella*
P2559S,P2559Y	*Croceibacter*
Pf-WMP4, PSS2, P-SSP7	*Cyanobacteria*
RC-2014	*Dickeya*
phiKT, ADB-2, Phax-I, vB_EcoM_CBA120,ECML-4	*Escherichia*
SH1	*Haloarcula*
JM-2012	*Halocynthia*
phiHAP-1	*Halomonas*
HF2	*Halorubrum*
1961P, KHP30, KHP40	*Helicobacter*
SC1	*Liberibacter*
psiM2	*Methanobacterium*
PhiCh1	*Natrialba*
P-HM1, P-HM2, pRSM4, P-SSM2, P-SSM4, PSSM-7, Syn1, Syn33	*Prochlorococcus*
PM2, h105/1	*Pseudo-alteromonas*
KPP10, lu11, Luz24, PA11, PaP3, Tf, TL	*Pseudomonas*
RSB2, RSL-1	*Ralstonia*
16–3	*Rhizobium*
RcapMu	*Rhodobacter*
Rm378	*Rhodothermus*
vB_SalM_SJ2, Maynard, Vi01, SFP10, vB_SalM_SJ3	*Salmonella*
Spp001, 1/4	*Shewanella*
phiSboM-AG3	*Shigella*
P60, S_CBS4, S-PM2, SRS-M4, S-ShM2, S-SM1, S-SM2, S-SSM5, S-SSM7, Syn19, Syn5, Syn9	*Synechococcus*
BA3	*Thalassomonas*
IN93, P23–45, P23–77, P74–26, phiYS40	*Thermus*
cp-t, phi-A318, SHOU24, SIO-2, vB_VchM-138, VP2, VP5, VP882, VP93	*Vibrio*

Normally, absent a lysis cluster, searching for spanins is most readily done by first looking for a lipoprotein gene, which are rare in phage genomes and encode the distinctive N-terminal lipobox signal. In 31 genomes, a lipoprotein gene was identified without a closely linked i-spanin candidate (type II integral membrane proteins). For example, Acinetobacter phage vB_AbaM_Acibel004, had gene 41 encoding a lipoprotein, but we could not detect any putative i-spanin gene in the vicinity. Genes encoding type II IM proteins, which could theoretically take up the role of an i-spanin, could be found in other locations. The most extreme case of separation in two-component spanins identified in this study was seen in the phage B40–8, where the predicted i-spanin and o-spanin are separated by ~ 1 kb. However, if there were any phages where the i-spanin and o-spanin were separated by a greater genomic distance, they could not be identified through our current standards.

Other than poor annotation or physical separation of the i-spanin and o-spanin genes, another potential reason for the absence of spanin genes in some phages is that the OM disruption is not necessary for phage lysis in these cases. This could result from differences in the organization and stability of the cell envelope and/or the natural environment of the host. For example, 20 of the 91 phages with no identifiable spanins were isolated on either *Prochlorococcus* or *Synechococcus* hosts. These marine cyanobacteria are usually found in the euphotic zone in the oceans, where the cells experience extreme physiological conditions [50]. The OM of these hosts might be fragile in those environments, such that the disruption of the IM and PG by the holin and endolysin is sufficient for lysis.

Among the 91 genomes where we could not identify a spanin gene, 34 had no CDS potentially encoding a lipoprotein. This included a number of phages with large genomes, within which it would be impractical to analyze every possible reading frame. Taking 50 kb as an upper limit, we analyzed 16 of the no-spanin genomes with the LipoSearch tool [51], which inspects for potential lipoproteins in every possible reading frame, irrespective of the annotated gene structures. Seven genomes were unambiguously devoid of a lipoprotein coding sequence in any reading frame, even considering potential frame-shift mutations, thus ruling out the presence of either an o-spanin or u-spanin gene. For example, *Acinetobacter* phage Petty has identifiable holin and endolysin genes of the canonical type, but no lipoproteins at all in any reading frame [52]. It can therefore be deduced that these phages employ a different mode of OM disruption. A possible alternative for OM disruption can be derived from recent reports that characterized phage endolysins with membrane penetrating activity [53, 54]. A highly positively charged C-terminal domain with predicted amphipathic helices was a common feature of these membrane-active endolysins, found in ϕKMV-like phages. Our present criteria also do not consider the possibility of proteins with other membrane topologies or anchors, to operate as spanins. For example, a type I (N-out, C-in) IM protein with a C-terminal TMD or an IM lipoprotein could also act as an i-spanin, while an OMP could act as an o-spanin.

Further experiments are needed to understand the mode of OM disruption in these spanin-less phages. A reasonable approach would be to shortlist phages that infect genetically facile hosts like *E. coli*, *Pseudomonas or Salmonella* and examine their lysis phenotype at the cellular level using phase contrast microscopy. If the phage shows no spanin lysis defect, plasmid libraries containing random segments of the phage genome can be constructed and screened for complementing the spanin lysis defect in the well-established lambda lysogen platform.

## Conclusion

For most phages, spanins are required to complete the last step of bacteriophage lysis; i.e. OM disruption. They play a key role in efficiently liberating progeny virions by causing rapid and complete breakdown of the OM, through a pathway that we have proposed to involve IM-OM fusion [10]. In this report, we identified spanins in more than 85% of the phage genomes and added new members to the existing set of two-component and unimolecular spanins. The SpaninDB established here will provide the framework for future spanin identification, classification and characterization. The findings of this research not only provide insights into spanin function, evolution, and domain organization but also the role of intermolecular disulfide bonds in stabilizing the spanin complexes and completing membrane fusion. For phages with no identifiable spanins, hypotheses for other possible modes of OM disruption were suggested. The results suggest several new questions and opens avenues for genetic and biochemical experiments. For example, the mechanism of u-spanin function can be addressed by a spheroplast fusion assay, like the one used for probing the ability of two-component spanins to fuse membranes. Small peptides designed based on the ectodomains of spanins which could be potential inhibitors of spanin function, can be titrated into the spheroplast fusion assay to manipulate the timing and efficiency of fusion [55, 56], which can have huge practical implications. Structural studies of spanins would help in understanding the subdomain organization in different spanins and different conformations the spanin complexes assume during different stages of the membrane fusion pathway. They would also shed light on the fundamental question of how u-spanins and two-component spanins approach the same solution even though being totally different in secondary structure compositions. All these suggested future studies using spanins can have serious implications for understanding the cellular envelope of Gram-negative bacteria, and the attributes of OM in particular. Understanding the mechanistic details of spanin function can also pave way for developing biotechnological applications like fusion assays, cargo targeting and delivery systems etc. as well as medical applications such as antimicrobial strategies.

## Methods

### Bioinformatics procedures

Identification of spanins and implications for automated phage annotation was done as described in Additional file 1: Figure S1. All the following information for every phage genome with an identified spanin system was added manually to the SpaninDB; phage name, accession version, architectural class (embedded/overlapped/separated), host, spanin GI (Geneinfo Identifier) number, spanin gene name or coordinates, predicted Shine-Dalgarno sequence for the spanin, primary structure of the spanin, periplasmic domain sequence of the spanin, length of the spanin, number of the periplasmic cysteines in the spanin, lipobox sequence for o-spanin/ u-spanin. All protein sequence and statistical analysis was done using tools on the CPT Galaxy platform (https://cpt.tamu.edu/galaxy-pub/). In brief, FASTA libraries of spanin sequences were created based on architecture and spanin type. These FASTA libraries were used to group the spanins into families using the BLASTCLUST tool. The length coverage threshold and score coverage threshold cutoffs were set to 0.4 and 40 respectively. These parameters were chosen because they gave us the optimal fit between finding distantly related sequences and minimalizing the false positive hits within each family. The families resulting from these parameters were also consistent with the families from our previous report on Rz-Rz1 equivalents [19]. These parameters were changed to 1 and 100 respectively to find identical spanin sequences. Manual corrections to the BLASTCLUST results were done using the online BLASTp tool (https://blast.ncbi.nlm.nih.gov/Blast.cgi) following the same threshold parameters. Sequence alignments were performed using the desktop version of CLC Main Workbench 7.6.2 (gap open cost = 10.0 and gap extension cost = 1.0). We used a “non-identical set”, in which only one sequence was chosen to represent all of its duplicates (Table 6), for secondary structure analysis and coiled-coil predictions. Secondary structure analysis was done using Jpred4 [57] (http://www.compbio.dundee.ac.uk/jpred/) and coiled-coils were predicted using Pepcoil with default settings [58] (http://www.bioinformatics.nl/cgi-bin/emboss/pepcoil). The consensus sequence for the lipobox was plotted using the logo generator software Webb Logo 3.3 [47] run locally on the CPT Galaxy instance. All data was plotted using the graphing and analysis software Kaleidagraph.

### Bacterial strains, culture growth, plasmid constructions and general methods

All cloning procedures were performed using the strain XL1-Blue *recA1 endA1 gyrA96 thi-1 hsdR17 supE44 relA1 lac* [F′ *proAB lacIqZΔM15 Tn10*] (Stratagene). For the complementation experiments, the temperature sensitive lysogen MC4100 (λ*cI857 Rz*_*Q100am*_*/Rz1*_*W38am*_) [7] carrying the indicated pRE plasmid derivatives was used. The pRE plasmid, a pBR322 derivative, contains the phage λ late promoter pR′ located directly upstream of a multiple cloning site and can be thermally induced. The p*pseT.3pseT.2* plasmid was constructed by replacing the *RzRz1* embedded cassette in p*RzRz1* [8] with the separated *pseT.3* and *pseT.2* genes between the KpnI and BamHI restriction sites. The construction of p*gp11* plasmid used for *gp11* complementation studies has been described earlier [19]. Site directed mutagenesis was used to introduce the cysteine mutations into *pseT.2* and lipobox mutations into *gp11*. Oligonucleotides were purchased from Integrated DNA Technologies, Inc. (Coralville, IA). Site directed mutagenesis was confirmed by sequencing at Eton Biosciences. The sequences of the primers used for all PCR amplifications and site-directed mutagenesis are available on request. Cultures were grown in LB medium at 30°C supplemented with appropriate antibiotics, ampicillin (Amp; 100 μg/ml) or kanamycin (Kan; 30 μg/ml) and 10 mM MgCl_2_ and induced for lysis by shifting to 42°C for 15 min before shifting to 37°C until the end of the experiment. A_550_ of the cultures was followed using the Gilford Stasar III spectrophotometer.

### SDS-PAGE and Western blotting

SDS-PAGE and western blotting were performed as described previously [59]. TCA pellets were washed in two volumes of acetone, resuspended in 1X SDS-PAGE buffer with or without β-mercaptoethanol (100 mM BME) and processed as indicated. Protein samples were loaded onto 10% resolving Tris-tricine polyacrylamide gels after loading volumes were normalized according to A_550_ at the time of collection of TCA precipitates. Proteins were transferred to PVDF membrane (Pall Life Sciences) using a Hoefer TE unit at 0.1 mA overnight at 4 °C. Antibodies (Sigma Genosys) were generated in rabbits against the synthetic peptides CERENEKLRKDAKKA, corresponding to the PseT.3 residues 74–87 and CWLNDVKRYVHDQKT, corresponding to the PseT.2 residues 71–84. The primary antibodies were used at a dilution of 1:1000 while the secondary antibody, goat-anti-rabbit-HRP (Thermo Scientific), was used at a dilution of 1:5000. Chemiluminescence was detected using a Bio-Rad XR Gel Doc system. SeeBlue Plus2 (Invitrogen) pre-stained standard served as a molecular mass standard.

## Additional files

Additional file 1: **Figure S1.** Flowchart showing the manual search protocol to identify potential spanin candidates from genomes of phages infecting Gram-negative hosts. All the corrected CDS collected from the phage genome were run through TMHMM 2.0 and LipoP 1.0 with default parameters. Any CDS from the output with an N-terminal TMD or a C-terminal TMD or an N-terminal lipoylation signal sequence were further investigated as described. This manual search was supplemented by the automated FindSpanin workflow on the CPT Galaxy instance [32]. Once a spanin candidate was confirmed and curated, it was added to the online SpaninDB [33] and served as a query to find other potential candidates using BLAST. (PDF 77 kb)

Additional file 2: **Table S1.** A table containing information about (a) two-component spanins (2CS) and (b) unimolecular spanins identified and analyzed in this study. It also serves as the initial basis for a continuously updated SpaninDataBase (SpaninDB) at the Center for Phage Technology website [33]. (XLSX 445 kb)

Additional file 3: **Table S2.** A table containing data from secondary structure predictions from the Jpred tool for (a) i-spanins, (b) o-spanins and (c) u-spanins. (XLSX 76 kb)

Additional file 4: **Table S3.** A table containing data from coiled-coil predictions from the Pepcoil tool for (a) i-spanins, (b) o-spanins and (c) u-spanins. (XLSX 86 kb)

Additional file 5: **Table S4.** A table showing different information about gene arrangement of o-spanin with respect to i-spanin for the embedded, overlapped and separated spanins. (XLSX 124 kb)

Additional file 6: **Table S5.** A table showing (a) number of periplasmic cysteines in 2CS (b) position analysis of cysteines in periplasmic domain of the entire spanin complex (c) position analysis of cysteines in periplasmic domain of i-spanins (d) position analysis of cysteines in periplasmic domain of o-spanins (e) position analysis of cysteines in periplasmic domain of the entire spanin complex for 2CS with only one periplasmic cysteine in either i- or o-spanin. (XLSX 656 kb)

## Abbreviations

2CS: Two-component spanins
CC: Coiled-coil
CPT: Center for Phage Technology
IM: Inner membrane
OM: Outer membrane
PG: Peptidoglycan
PMF: Proton motive force
PRR: Proline-rich region
SAR: Signal Anchor Release
SpaninDB: Spanin database
TMD: Transmembrane domain
U-spanin: Unimolecular spanin
