# Supplementary figures and images for: Phage spanins: diversity, topological dynamics and gene convergence
Source: BMC Bioinformatics. 2018 Sep 15;19:326. doi: 10.1186/s12859-018-2342-8 (PMC6139136; doi:10.1186/s12859-018-2342-8)

**S1**

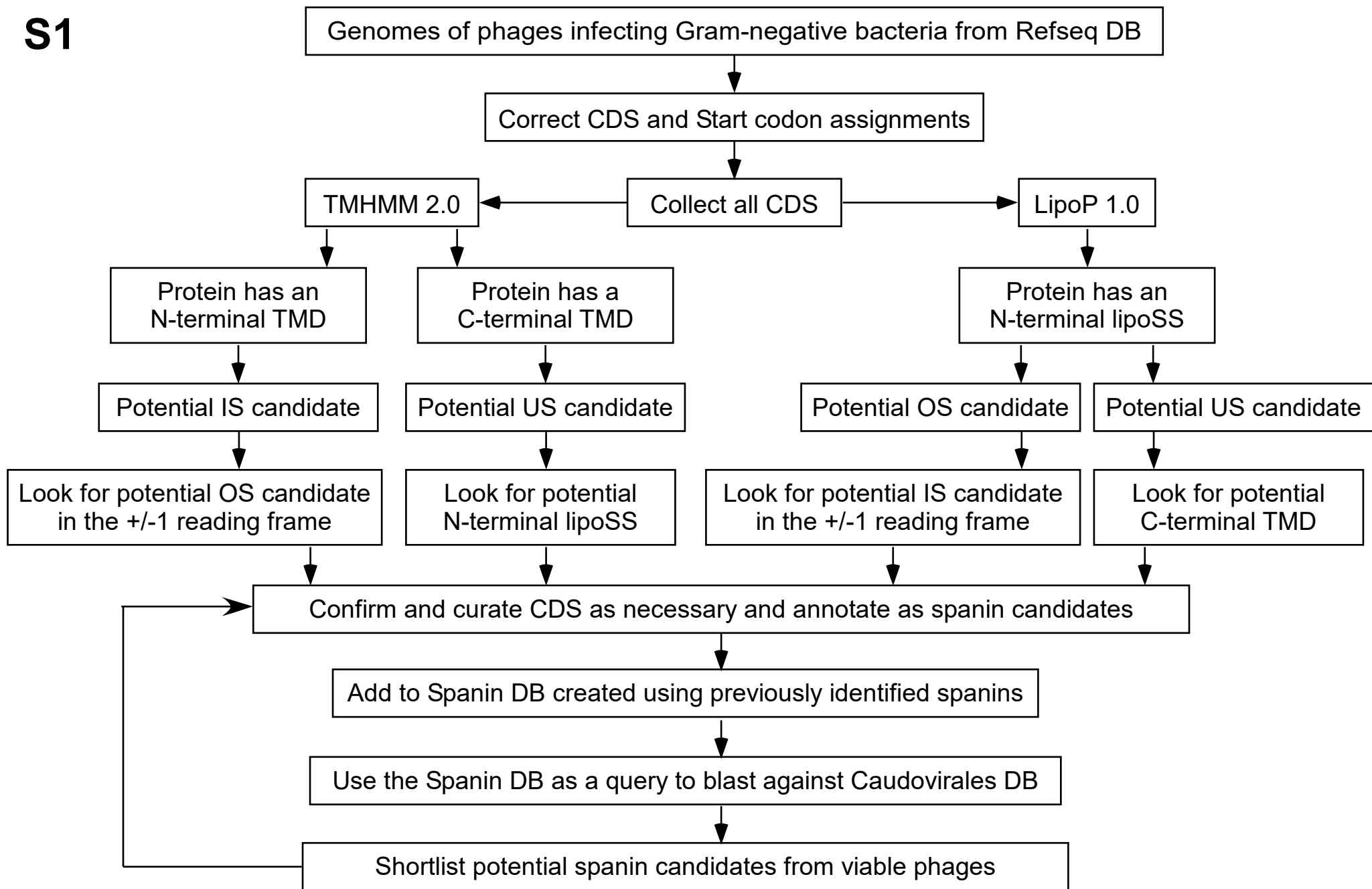

Supplement: Supplementary file 1 — Figure S1. Flowchart showing the manual search protocol to identify potential spanin candidates from genomes of phages infecting Gram-negative hosts. All the corrected CDS collected from the phage genome were run through TMHMM 2.0 and LipoP 1.0 with default parameters. Any CDS from the output with an N-terminal TMD or a C-terminal TMD or an N-terminal lipoylation signal sequence were further investigated as described. This manual search was supplemented by the automated FindSpanin workflow on the CPT Galaxy instance [32]. Once a spanin candidate was confirmed and curated, it was added to the online SpaninDB [33] and served as a query to find other potential candidates using BLAST. (PDF 77 kb) [file 12859_2018_2342_MOESM1_ESM.pdf]
